# LitChemPlast: An Open Database of Chemicals Measured in Plastics

**DOI:** 10.1021/acs.estlett.4c00355

**Published:** 2024-10-29

**Authors:** Helene Wiesinger, Anna Shalin, Xinmei Huang, Armin Siegrist, Nils Plinke, Stefanie Hellweg, Zhanyun Wang

**Affiliations:** † Chair of Ecological Systems Design, Institute of Environmental Engineering, ETH Zürich, 8093 Zürich, Switzerland; ‡ National Centre of Competence in Research (NCCR) Catalysis, Institute of Environmental Engineering, ETH Zürich, 8093 Zürich, Switzerland; ¶ Department of Earth Sciences, University of Toronto, Toronto, Ontario M5S 1A1, Canada; § Department of Environmental and Occupational Health Sciences, University of Washington, Seattle, Washington 98195, United States; ∥ Laboratory of Sustainable Food Processing, Institute of Food, Nutrition and Health, ETH Zürich, 8092 Zürich, Switzerland; ⊥ Empa - Swiss Federal Laboratories for Materials Science and Technology, Technology and Society Laboratory, 9014 St. Gallen, Switzerland

**Keywords:** Use Patterns, Circular Economy, Targeted Measurements, Nontargeted Screening, Chemicals in Plastics, Substance Flow Analysis, Exposure Modeling

## Abstract

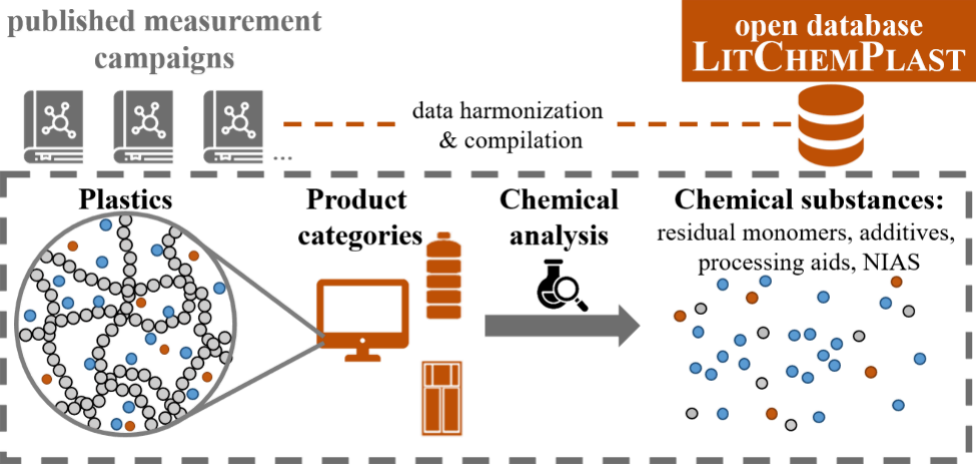

Plastics contain various chemical substances, which can impact human and ecosystem health and the transition to a circular economy. Meanwhile, information on the presence of individual substances in plastics is generally not made publicly available, but relies on extensive analytical efforts. Here, we review measurement studies of chemicals in plastics and compile them into a new LitChemPlast database. Over 3500 substances, stemming from all plastic life-cycle stages, have been detected in different plastics in 372 studies. Approximately 75% of them have only been detected in nontargeted workflows, while targeted analyses have focused on limited well-known substances, particularly metal(loid)s, brominated flame retardants, and *ortho*-phthalates. Some product categories have rarely been studied despite economic importance, e.g., consumer and industrial packaging (other than food packaging), building and construction, and automotive plastics. Likewise, limited studies have investigated recycled plastics, while existing measurements of recycled plastics show higher detection frequencies and median concentrations of regulated brominated flame retardants across many product categories. The LitChemPlast database may be further developed or utilized, e.g., for exposure assessment or substance flow analysis. Nonetheless, the plethora of relevant substances and products underscores the necessity for additional measures to enable the transition to a safe circular plastics economy.

## 1 Introduction

Plastics are widely used across various product categories, e.g., packaging, building and construction (B&C), or household items, with the growing global annual production reaching 460 megatonnes in 2019.^1,2^ Many environmental impacts (such as greenhouse gas emissions, micro- and macroplastic pollution) and human health impacts are associated with their production, use, disposal, and recycling, and are projected to increase in the near future.^1,3,4^ For example, plastic production has shifted to more coal-based economies in recent years, which has increased the associated greenhouse gas emissions.^4^

Plastics can contain numerous chemical substances, including residual monomers from polymer production, additives to impart specific properties, processing aids, and nonintentionally added substances (NIASs) such as contaminants, byproducts, and/or degradation products.^5−9^ Recent studies have jointly identified more than 16 000 substances that are associated with plastics and plastic products.^10−13^ More than 4 200 of these substances are hazardous, meaning that they are persistent, bioaccumulative, mobile, and/or toxic.^13^

These substances are typically not chemically bound to the polymer matrix and may be released throughout the entire plastic life cycle. This can lead to exposure of both humans and the environment, and result in adverse effects on both.^14−26^ Estimating the release of and exposure to these substances requires concentration information of chemicals in mixtures, plastic products, and waste.^27−29^ In addition, mechanical recycling, which can reduce several environmental impacts of plastics over multiple life cycles,^30−32^ still faces challenges associated with input materials, including quality constraints and the presence of hazardous substances.^30,32−34^ Many substances present in the input materials can interfere with the recycling process and/or impact the safety and quality of recycled materials.^35−39^ Thus, understanding the presence and concentrations of (hazardous) substances is crucial for ensuring a safe and sustainable circular plastics economy.^34,40^

Currently, information on the identification, presence, and concentrations of chemicals in products and waste streams is largely unknown or scattered. In certain cases, information disclosures are mandated: for example, in the European Union (EU), the chemicals used in food-contact materials are limited to a positive list,^41^ and the intentional inclusion of Substances of Very High Concern (SVHCs) in products above 0.1 weight% has to be reported in the EU SCIP database.^42,43^ Meanwhile, for most plastics, producers do not need to provide information on intentionally added substances,^44^ and this information is thus not typically transmitted along the supply chain.^10−12^ Furthermore, no systematic understanding of NIASs in individual plastics exists.^7^ Thus, for most plastics, the presence and concentrations of substances can only be determined through chemo-analytical measurements. While numerous scientific studies have conducted these measurements, a comprehensive and consolidated overview of the results is lacking.

This study conducts a thorough review of chemo-analytical studies between 1978 and 2021 to establish an open database of chemicals measured in plastics, the LitChemPlast database. This database may be further built upon by the scientific community and other interested stakeholders. Here, we present a summary of existing measurements across various plastics, including information on the related product categories and the measured chemicals. To further illustrate potential uses of the database, we conduct two case studies: (1) using the data to understand the potential effects of mechanical recycling on the chemical composition, including the potential sources of contaminants in recycled plastics; and (2) discussing how the data may be used as inputs for substance flow analyses, exposure assessments and other uses. Finally, implications for future safe and sustainable recycling, and future development options of the database, are outlined.

## 2 Methods and Materials

The objective of this database was to map chemo-analytical measurements of chemicals in plastics and to compile their data in a comprehensive manner. Studies reporting the measured chemical composition of plastic raw materials (e.g., granulate, nurdles), products (e.g., agricultural, B&C, and packaging plastics; see Table S1 in the Supporting Information 1, SI1), and waste were considered relevant. The database includes studies from 1978 to 2021, identified using a keyword search on Web of Science and SciFinder, followed by a two-stage screening process (for details on the data sources, keywords, and screening process and criteria, see Section S1.2 in SI1). Studies on food packaging and textiles were not specifically searched for, but they were included if encountered. This is because comprehensive analyses and databases of chemicals in food packaging plastics are already available from others.^45−47^ Textiles are largely irrelevant for mechanical recycling and it is difficult to distinguish chemicals added to the end products from those in the plastic itself.^48,49^ It should be noted that this study is a scoping review, and did not specifically assess the quality of each study.

**Table 1:** Available Information in the LitChemPlast Database

Type	Specific Entries
bibliographic data	- first author
	- year
	- title
	- DOI
	- abstract
analyzed samples	- product (sub)categories
	- product name
	- polymer type
	- recycling status
sampling method	- sampling stage
	- sampling country
	- sampling year
	- sampling size
	- sampling procedure
analytical methods	- sample preparation
	- analytical method
	- nontargeted workflow
measured chemicals	- substance category
	- abbreviation
	- substance name
	- IUPAC name
	- CASRN
	- InChI
	- SMILES
measurement results	- LOD or LOQ [mg kg^–1^]
	- detection frequency [%]
	- concentration [mg kg^–1^]

Various pieces of information were manually extracted from each relevant study, including the bibliographic data, the analyzed samples (including their product categories and polymer types), the sampling and analytical methods, and the examined chemicals and their respective concentrations (Table 1).

To harmonize the information, chemicals were assigned their Chemical Abstracts Service Registry Number (CASRN) and a “substance category”, based on commonly targeted substance groups (Section S1.3.2 in SI1). Samples were grouped into hierarchical product categories, encompassing eight main categories (generic, packaging, B&C, automotive, electrical and electronic equipment or EEE, agriculture, textiles, and others) and 51 subcategories, as detailed in Section S1.3.2 and Table S1 in SI1, based on the detailed categories from the mass flow analysis by Klotz et al. (2022).^32^ Samples were also categorized by their polymer type (15 types, Table S2 in SI1), sampling stage (5 stages, Table S3 in SI1), sampling procedure (4 procedures, Table S4 in SI1), and recycling status. The recycling status was assigned, based on the explicit descriptions in the original sources, as either “recycled” or “virgin”, otherwise “not specified” was assigned. The analytical workflows in studies were categorized as either “targeted analysis” or “non-targeted screening”. In targeted analyses, the focus is on specifically detecting and often quantifying preselected compounds. In contrast, nontargeted screenings aim to comprehensively identify known and unknown chemicals present in a sample. The concentration data were harmonized to units of milligrams of chemical per kilogram of plastic (mg kg^–1^), whenever possible. Data points that could not be expressed in these terms were excluded from the final database. All harmonized information was then fed into the LitChemPlast database, which is provided as SI2 in the format of Excel Spreadsheets. All analyses and graphs were created using Python, with the Jupyter notebook provided in SI3.

## 3 Overview of the Data

Overall, 786 studies have been downloaded and the full paper was reviewed. Subsequently, measurement data have been extracted from 372 studies, using targeted (*n = 286*), nontargeted (*n = 61*) and mixed (*n = 25*) workflows. Around 67% of the studies have been conducted since 2010, while some individual studies may be as early as 1978 (Figure S3 in SI1). In total, more than 47 000 samples have been tested and more than 3 600 chemicals have been detected, resulting in over 65 000 entries in the LitChemPlast database.

### 3.1 Overview of the Samples

**Figure 1:**
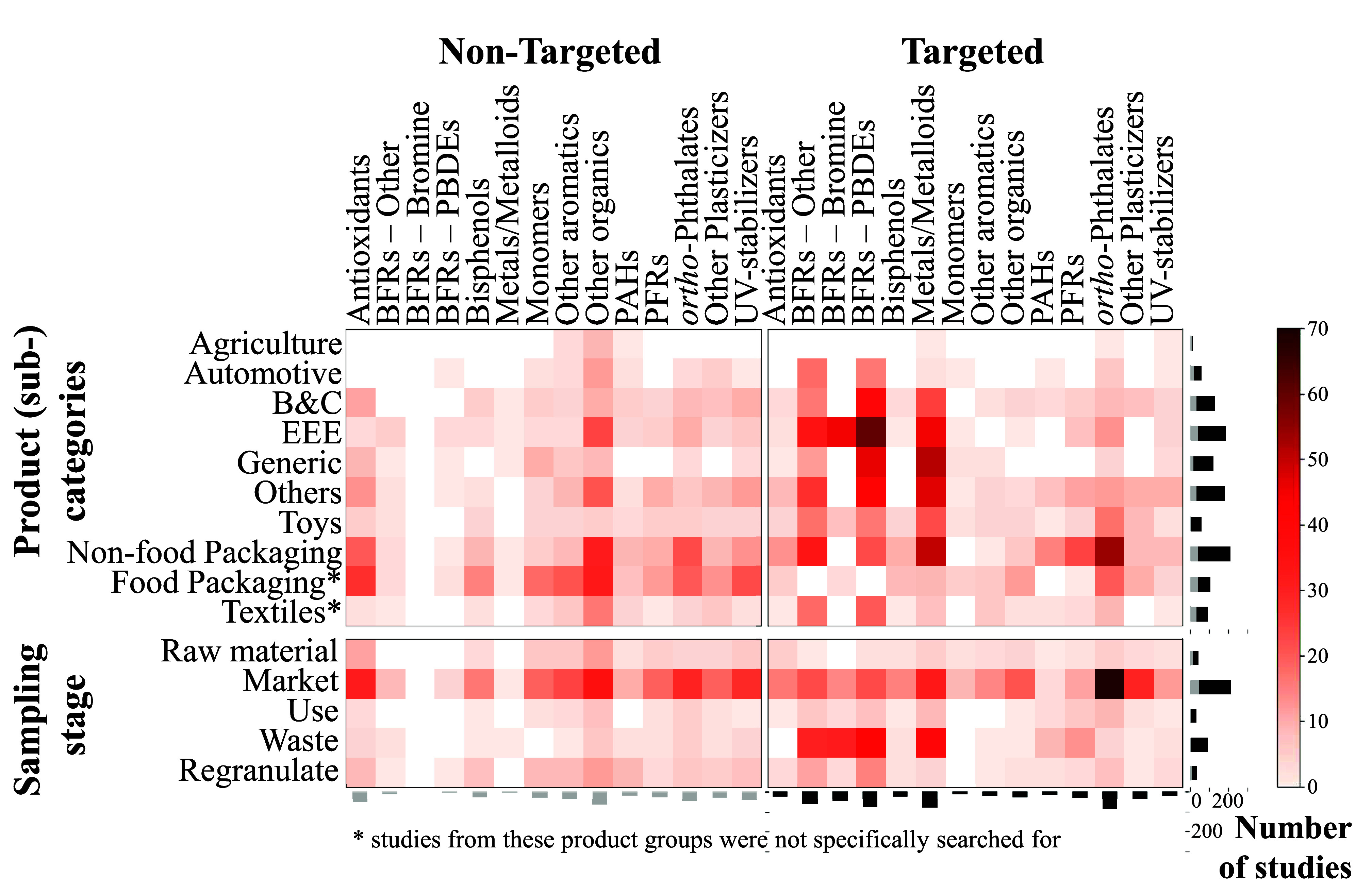
Number of targeted and nontargeted studies conducted per substance type, product (sub)category, and sampling stage. The light gray bars indicate the number of nontargeted studies, and the dark gray bars indicate the number of targeted studies. Note that studies on the product (sub)categories “food packaging” and “textiles” were not specifically searched for. BFRs = brominated flame retardants, PBDEs = polybrominated diphenyl ethers, PAHs = polycyclic aromatic hydrocarbons, PFRs = phosphorus flame retardants/plasticizers, UV = ultraviolet light, B&C = building and construction, and EEE = electrical and electronic equipment. “Generic” encompasses samples without a specific description, e.g., described as “raw material”, “plastic”, or “waste” (see Table S1 in SI1).

The product categories show vast differences regarding the number of studies conducted and samples analyzed. Several product categories have been frequently researched (Figure 1), for example, food packaging, EEE, household items, or toys. Although our study did not specifically search for studies on chemicals in food packaging, over 26% of the studies included focus on them. Generally, many studies concentrate on product categories with high exposure potential, such as food packaging, medical equipment, household products, or toys (Figure S18 in SI1). Furthermore, product (sub)categories with specific regulations are a common focus of the research, for example, food packaging (regulated in the EU under the food contact materials regulation^50^), or EEE (regulated in the EU under the Restriction of Hazardous Substances in Electrical and Electronic Equipment (RoHS) Directive 2011/65/EU^51^).

Targeted and nontargeted studies generally focused on different product categories (Figure 1). Nontargeted studies predominantly focused on (food) packaging, which is in line with the regulatory requirements in some regions demanding migration tests.^50^ Targeted studies lacked a clear sectoral focus, but compared to nontargeted studies, they tested more EEE, medical items, household items, and toys. This is also in line with regional regulatory requirements for these product (sub)categories or their waste, which prohibit the presence of specific substances or elements.^51,52^

Regarding the life-cycle stages, nontargeted studies concentrate on samples from the market stage, whereas targeted studies also sample from the waste stage. Meanwhile, there are significant gaps in the testing of virgin raw materials, regranulates, and products in use (Figure 1).

For many samples, the polymer types were not specified (for 2 476 of 4 873 sample entries, i.e., 51%, it was not reported) and just referred to as “plastic”. The studies that reported the polymer types mainly focused on the commercially important polymer types (i.e., PET, PP, HDPE, PS), with PET being the most commonly reported. Certain substances are predominantly tested in specific polymer types likely due to their expected use therein, e.g., *ortho*-phthalates are repeatedly targeted in PVC samples.^53,54^

Regarding the sampling location, 54% of all the studies collected samples in high-income regions, 23% of the studies did not specify the sampling location, and 14% sampled primarily in China and India. Thus, the studies conducted so far have poor regional coverage for low-income countries (3%, Figures S5 and S6 in SI1). The global trade and transport of plastic products and waste may reduce the impact of this data gap, as studies from high-income countries may include imported plastics or plastics bound for export in their measurement samples, and thus, these studies may also be relevant to other regions.

### 3.2 Reported Substances

Overall, 3 488 substances, identified by their active CASRNs, along with 57 elements and 160 trivial names or groups of chemicals (which could not be mapped to individual substances or CASRNs), have been detected in various plastics (see LitChemPlast, Sheet “Chemicals” in SI2).

**Figure 2:**
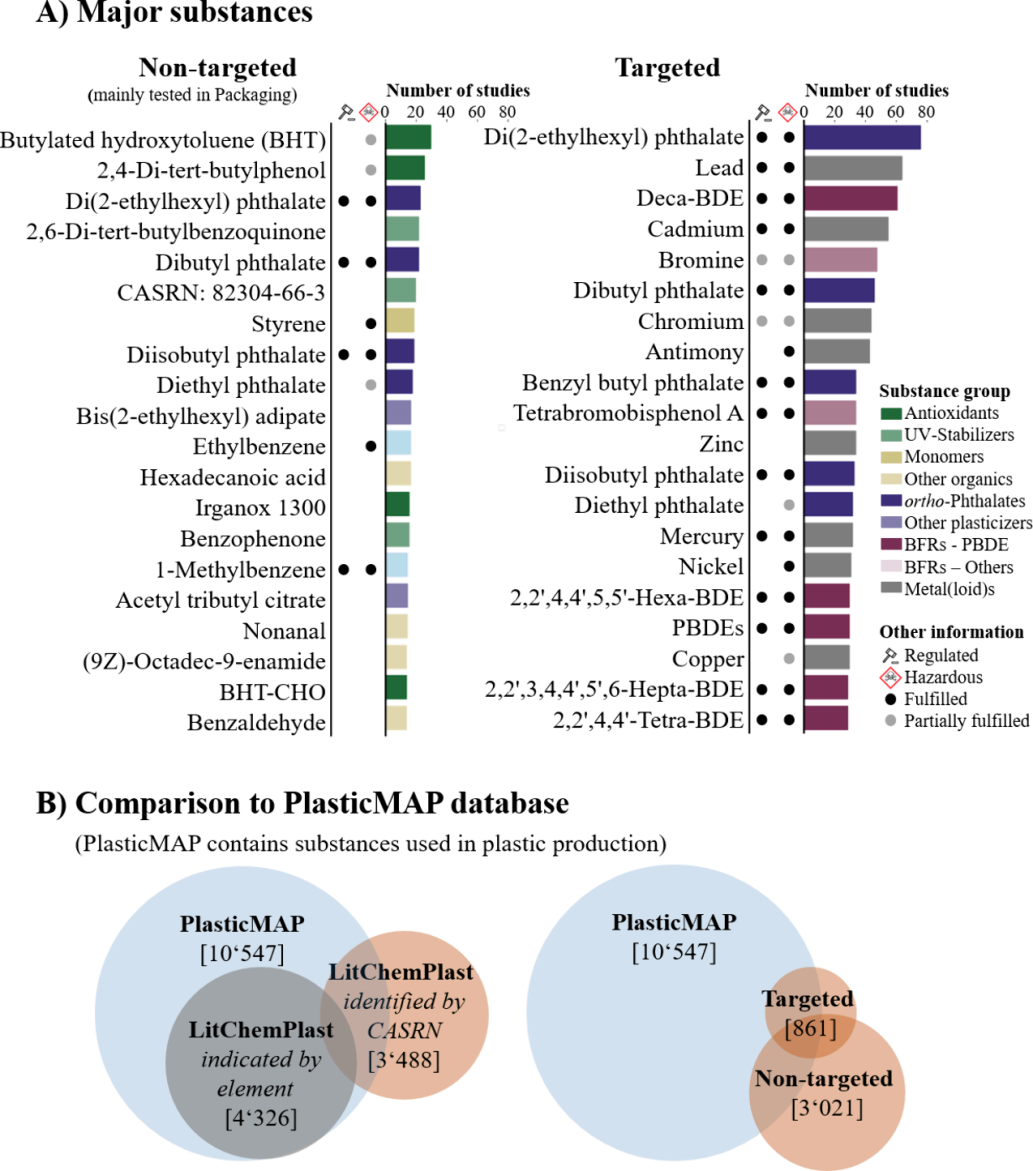
Overview of the substances detected in plastics in the LitChemPlast database. (A) Top twenty most frequently detected substances in nontargeted and targeted workflows. Further information on the top 20 targeted substances can be found in Figure S12 in SI1. (B) Overlap between the substances in the LitChemPlast database and the PlasticMAP database.^10^ Substances: CASRN: 82304-66-3 = 7,9-Di-*tert*-butyl-1-oxaspiro[4,5]deca-6,9-diene-2,8-dione; BHT-CHO = 3,5-Di-*tert*-butyl-4-hydroxybenzaldehyde (CASRN: 1620-98-0); BDE = brominated diphenyl ether.

The vast majority of individual substances (>75%) have only been detected through nontargeted workflows (Figure 2). It is important to note that while nontargeted approaches can identify a wide range of chemicals without prior knowledge, the range of detectable chemicals is limited by the sample preparation and treatment and the analytical methods used.^55^ In this database, nontargeted approaches relied on gas chromatography–high-resolution mass spectrometry (GC–HRMS) or liquid chromatography–HRMS (LC–HRMS). As a result, only a limited chemical space is covered and not all possible chemical components are included, especially high-molecular-weight substances and metals. With this in mind, the most commonly detected substances in nontargeted workflows are antioxidants, UV stabilizers, *ortho*-phthalates, alternative plasticizers, and some simple organic substances such as hexadecanoic acid (CASRN: 57-10-3) or nonanal (CASRN: 124-19-6). Furthermore, many substances from nontargeted screenings have been categorized as “other organics” (i.e., they are organic substances but do not fit into any of the common groups in targeted screenings, see Section S1.3.2 in SI1). The common presence of these substances in nontargeted analyses and the fact that they are rarely studied in targeted analyses suggests that many substances may be understudied and seldomly quantified, such as butylated hydroxytoluene (CASRN: 28-37-0), 2,4-di-*tert*-butylphenol (CASRN: 96-76-4), or 2,6-di-*tert*-butyl-1,4-benzoquinone (CASRN: 719-22-2) (Figure 2 – A).

The most commonly targeted and quantified substances are *ortho*-phthalates, brominated flame retardants (BFRs), and metal(loid)s, many of which are hazardous, with multiple been regulated (Figure 2, Figure 1).^51,56^ Furthermore, many of these commonly targeted substances/elements have been detected through screening using simple spectroscopic techniques, for example, using X-ray fluorescence (XRF) for bromine or metal(loid)s. Thus, their associated measurements are available for a wider range of product categories than for other substances (Figure 1).

We also compare the substances listed in the LitChemPlast database with those in the PlasticMAP database, our previously published database of chemicals used as monomers, additives, and processing aids in plastic production.^10^ When considering only the measured metal and metalloid elements, existing measurements cover more than 40% of the substances in PlasticMAP (gray circle in Figure 2 – B left). For instance, tin measured by XRF can correspond to 142 organotin compounds and tin salts in the PlasticMAP database (such as tributyltin chloride, dimethyltin dichloride, or tin tetrachloride). However, when looking at individual substances with the same CASRNs, there appears to be limited overlap (covering 33% of the substances in LitChemPlast, and 11% of the substances in PlasticMAP, see Figure 2 – B).^10^

Generally, the more frequently a substance is reported in the studies reviewed, the more likely it is to be present in the PlasticMAP database. The discrepancy between the LitChemPlast and PlasticMAP databases is wider for the substances that have been detected specifically using nontargeted workflows than those solely detected using targeted workflows. Specifically, of the 861 substances that have been identified using targeted methods, two-thirds are also included in the PlasticMAP database, while the remaining 289 substances are mainly comprised of other brominated flame retardant isomers (∼ 50), pesticides (∼ 30), and polychlorinated biphenyl congeners (PCBs, ∼ 30).

The visible discrepancies between the substances used in plastic production (i.e., in the PlasticMAP database) and those measured in plastics (i.e., in the LitChemPlast database) are likely due to the following reasons:

(a) Inclusion of NIASs in analytical studies: Most NIASs are not covered by the PlasticMAP database, but may be present in samples. For example, persistent organic pollutants (POPs) such as PCBs have been commonly targeted and detected, but are usually not intentionally added to plastics. Another example is degradation products of antioxidants, such as 3,5-ditert-butyl-4-hydroxyacetophenone (CASRN: 14035-33-7), which are formed over the lifetime of plastics.
(b) Analytical artifacts: Measurement artifacts or mistakes may be reported, despite substances not being present in the plastic itself. For example, GC–MS techniques may produce siloxane ghost peaks (depending on the column, inlet, or septa used), which, without blank correction, may be falsely attributed to the sample.^57−59^
(c) Difficulties in matching the discrete compounds showing up in measurements to the mixtures being used in the production: Many substances used in the production of plastics are not “discrete compounds”, but so-called “substances of unknown or variable composition, complex reaction products, or biological materials (UVCBs)”, mixtures, and polymers (around 25% of all the substances in the PlasticMAP database^10^). However, most chemo-analytical techniques may only detect discrete compounds. For example, most nontargeted MS approaches would tentatively assign a substance identity to individual peaks or molecular features, rather than checking if the measured combination may represent some mixtures. For example, the UVCB Hexamoll DiNCH may be present in samples, but only some individual isomers such as bis(7-methyloctyl) cyclohexane-1,2-dicarboxylate are typically measured.
(d) Lack of analytical methods and standards: Many substances present in the PlasticMAP database are not detectable using current analytical methods, as at least 5% of all these substances lack any commercially available standards, according to SciFinder.^10^

Furthermore, there may also be some gaps regarding intentionally added substances in the PlasticMAP database, especially for substances for which many isomers or congeners exist. For example, the major commercially relevant polybrominated diphenyl ethers (PBDEs) are part of the PlasticMAP database; however, several individual congeners that were present in the measurement studies are not part of the database.

## 4 Applications of the LitChemPlast Database

### 4.1 Understanding Potential Effects of Mechanical Recycling

#### 4.1.1 Comparison of Recycled and Virgin Plastics

Mechanical recycling of plastics can be an option for dealing with plastic waste, and can reduce the environmental impacts of plastics such as greenhouse gas emissions.^1,60^ However, mechanical recycling is challenged by the diversity of plastic products, composite materials (such as multilayer packaging), quality loss during recycling, and limited options for recyclate utilization.^30,32,33,61,62^ Furthermore, recycled plastics can be contaminated by hazardous chemicals, posing a risk to human and ecosystem health.^34^ To date, comprehensive research on this matter remains limited; this study has identified only 35 studies that have tested regranulates (Figure 1 – Regranulate). Most of these studies have focused on legacy additives, such as *ortho*-phthalates, brominated flame retardants, and metal(loids). Unfortunately, only a few studies have measured substances in virgin and recycled plastics of the same product (sub)categories. Thus, limited comparisons can be made. In addition, comparing the detection frequencies and measured concentrations across studies is complicated by the different study designs, sampling locations, sampling years, sampling procedures, sample preparation and treatment, and analytical techniques.

**Figure 3:**
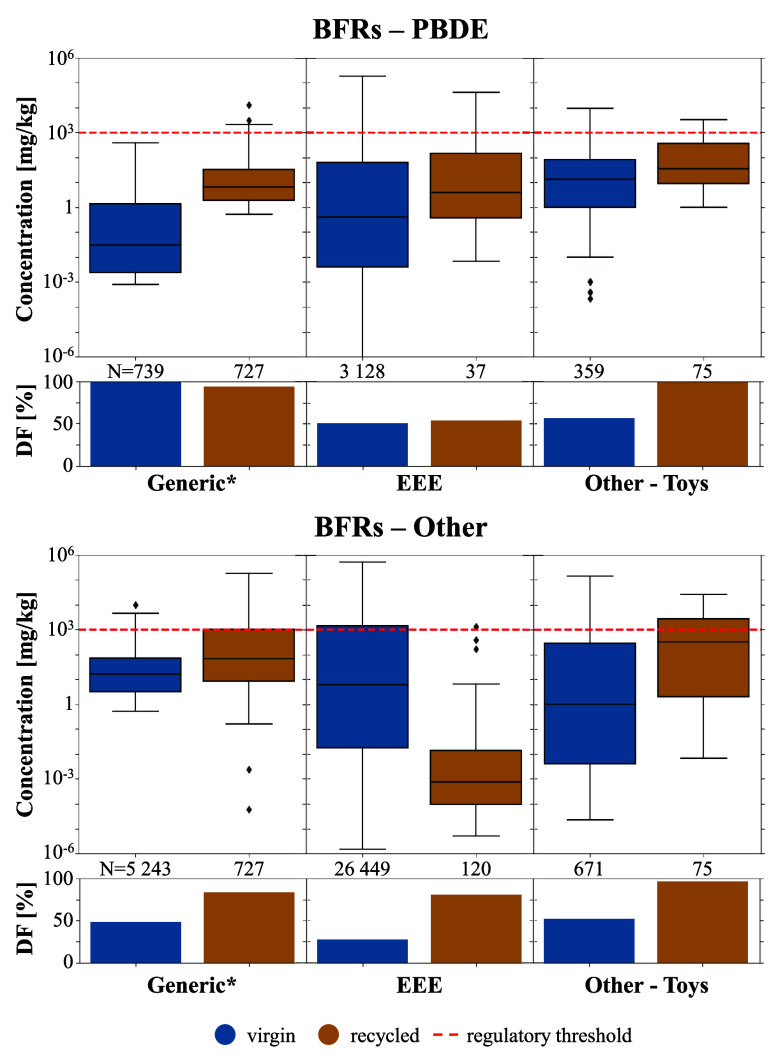
Comparison between recycled and virgin plastics for different product categories and substances in terms of the detection frequencies (DF) and concentration distributions (boxen plot). Only brominated flame retardants and selected product categories are displayed, as these are the only combinations for which sufficient measurements are recorded (>20 samples per group). The red line signifies a commonly used regulatory threshold of 1000 mg kg^–1^ as a benchmark. * “Generic” encompasses products that lacked a specific description, e.g., those samples described as “raw material”, “plastic”, or “waste” (see Table S1 in SI1).

Building upon the studies in the LitChemPlast database, brominated flame retardants in recycled and virgin plastics from generic plastics, EEE, and toys can be preliminarily compared (more than 20 samples per category were measured). Concentrations within a single product category can vary widely, spanning several orders of magnitude (e.g., from nondetects to 1 000 mg kg^–1^, see Figure 3) and typically deviate from a normal distribution. In general, both legacy and alternative brominated flame retardants show a higher detection frequency in recycled plastics than in virgin plastics. The results are mixed in terms of concentrations: (1) legacy substances such as PBDEs present higher concentrations in recycled plastics used in all the categories analyzed, while (2) alternative brominated flame retardants show higher concentrations in virgin EEE plastics (in which they are intentionally added for their first life cycle), but higher concentrations in recycled plastics of the other product categories. No temporal trend in recycled or virgin plastics is ascertainable (Section S2.3 in SI1).

Although a direct comparison of the samples is complicated by the varying sampling regions and years, these findings for brominated flame retardants are in line with the assumed pathway of legacy chemicals and their alternatives in the economy:^34^

(a) Legacy chemicals are expected to disappear from all virgin materials due to regulatory compliance, thus showing lower detection frequencies and concentrations. In contrast, in recycled materials, part of the “stock” of legacy chemicals is retained via recycling, resulting in higher detection frequencies and concentrations compared to virgin materials.
(b) Alternatives, on the other hand, are expected to be high in virgin materials in product categories where they are intentionally used, thus showing higher concentrations there. In the other product categories where they are not typically used, they should primarily be present via contamination during the production or via recycled materials, thus showing low detection frequencies and concentrations in virgin materials, but high detection frequencies and concentrations in regranulates, in these other product categories.

#### 4.1.2 Sources of Contamination in Recycled Plastics

The reviewed scientific literature on the chemical composition of recycled plastics has largely focused on well-known legacy additives that have been regulated, such as several *ortho*-phthalate plasticizers, brominated flame retardants, and toxic metals (Figure 1 – Regranulate; Table 2). The most commonly observed substances have been bromine and brominated flame retardants, antimony (a catalyst in PET polymerization), and bisphenol-A (BPA, CASRN: 80-05-7, a common monomer in polycarbonate production), all of which were likely intentionally added for their first application. Meanwhile, brominated flame retardants, likely originating from waste EEE, have been found in recycled household and consumer items as well as in B&C items.^63−70^ Antimony catalyst residues have been detected in PET bottle regranulates. BPA has been found in regranulates made from polycarbonate EEE plastics.^71^

**Table 2:**
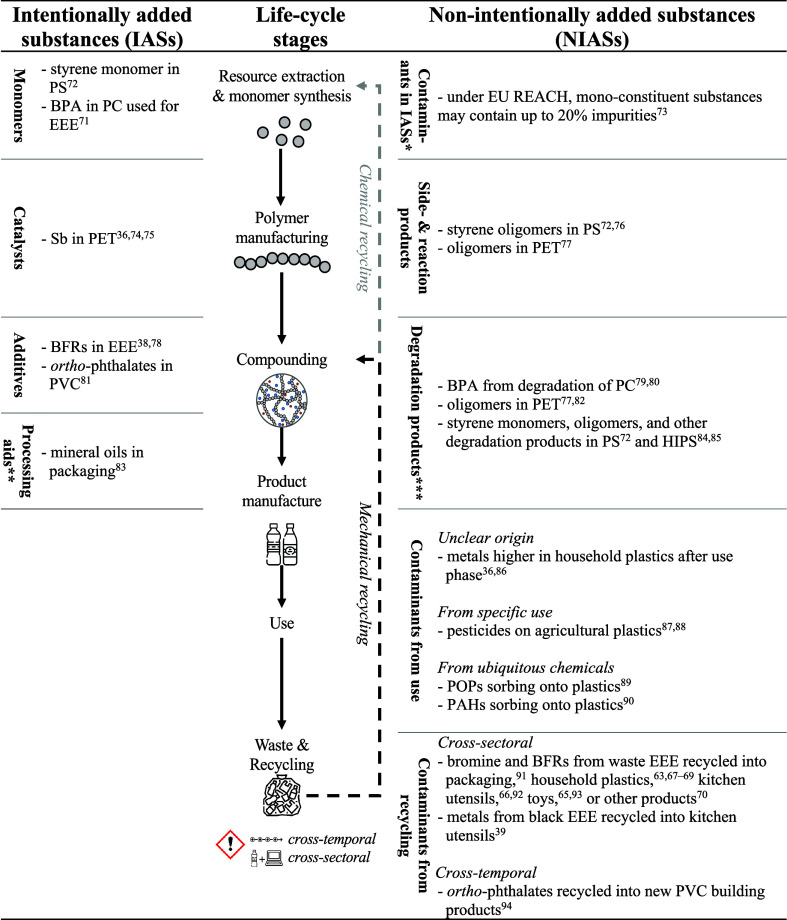
Sources of Chemicals in Plastics along Their Life Cycle with Examples from the Literature^a^

a PS = polystyrene, BPA = bisphenol A (CASRN: 80-05-7), PC = polycarbonate, EEE = electrical and electronic equipment, PET = poly(ethylene terephthalate), BFRs = brominated flame retardants, PVC = poly(vinyl chloride), HIPS = high-impact polystyrene, POPs = persistent organic pollutants, and PAHs = polycyclic aromatic hydrocarbons.

* Contaminants may be contained in all IASs, not just monomers.

** Processing aids may also be added during previous life-cycle stages.

*** Degradation may also happen during later life-cycle stages.

The presence of such hazardous substances is of particular concern for:

1. Cross-sectoral recycling (i.e., the waste comes from another product (sub)category than the one in which the regranulates are used). Substances may end up in applications where their original function is not needed, but where they may lead to higher exposure and/or exposure of more susceptible populations. Consequently, they do not provide benefits in this new application. An example of contamination by cross-sectoral recycling is the recycling of brominated flame retardants from EEE waste into children’s toys.^65,95^
2. Cross-temporal recycling (relevant for product categories with long lifetimes, for which waste is generated long after the initial formulation). Substances that were commonly added in the past may no longer be considered safe, but may still be present in current and future waste streams. An example of contamination by cross-temporal recycling is the closed-loop recycling of *ortho*-phthalates or lead in PVC building materials.^94,96^
3. Accumulation of hazardous substances during reformulation. Due to the repeated addition, specific substances may accumulate over time, and thus, cause more exposure than expected. For example, the addition of certain additives is common during recycling, including compatibilizers and stabilizers.^97^ However, their (potential) accumulation over several life cycles has, to our knowledge, not been investigated in practice.

Meanwhile, chemicals contained in regranulates are considerably more diverse than the aforementioned ones. The contaminants may originate from multiple stages of the plastic life cycle, such as raw material production, polymerization, product manufacturing, use, and recycling, and may result from intentional addition, contamination, degradation, and side-reactions (for more details, see Table 2).

In particular, NIASs have rarely been studied and even less frequently been the target of research (Table 2).^98^ The majority of NIAS-related studies have focused on monomers or oligomers (note that it remains debated whether monomers should be classified as NIASs or not; nevertheless, they may be residuals from polymerization, and thus be considered as intentionally added, or they may be degradation products of the polymer during its life cycle, and thus, be considered as nonintentionally added).^7^ For example, recycled polycarbonates commonly contain the monomer BPA and various oligomers.^79,80^ Recycled styrenics (e.g., HIPS, expanded PS) commonly contain their monomer styrene (CASRN:100-42-5) and various oligomers.^72,84,85^ Recycled PET has been reported to contain primarily monomers, oligomers, and various reaction products (e.g., oxidation products).^77,82^ Several studies have also reported contamination from the use phase, including ubiquitous POPs such as PCBs,^89,90^ pesticides in agrochemical packaging,^87,88^ or various other contaminants of unknown origin in PET bottle regranulates.^86^ Therefore, the source(s) of NIASs may be difficult to determine when only looking at recycled plastics.

More importantly, current research does not comprehensively cover recycled plastics (Figure 1). There are significant research gaps concerning studies on (1) degradation products of polymers other than styrenics, polycarbonates and PET, especially for those products that are exposed to harsh conditions (e.g., heat, UV radiation), (2) degradation products of high-volume additives, (3) plastics that are likely to be contaminated during the use phase (e.g., packaging of chemicals, automotive shredder residue^99^), or (4) common environmental pollutants other than POPs or pesticides.

Furthermore, the initial contamination of intentionally added substances may also be a source of contamination, but it is often overlooked. For example, the definition under the EU REACH regulation allows a monoconstituent substance to contain up to 20% impurities.^73^

Due to the rapidly evolving landscape of plastics production, recycling, and regulations, many developments may not be anticipated by past measurements. Thus, continued efforts to monitor the chemical content of plastics from all life-cycle stages is crucial.^100^

### 4.2 Inputs for Substance Flow Analysis, Exposure Assessment, and Other Uses

Substance flow analysis (SFA) is a method in industrial ecology that aims to provide relevant information on the presence, fate, transport and management of substances within a defined system.^101^ In general, the information needed to conduct an SFA depends on the system boundaries and the study goal.^101^ The LitChemPlast database can serve as an information resource for SFA practitioners for those systems in which plastics play a relevant role, for example, for understanding chemical flows under various plastic waste management options. We provide not only the concentrations of specific substances within plastic samples but also SFA-relevant details about these samples (e.g., polymer type, fine-grained product (sub)categories) and details that may help to assess the information quality (e.g., sampling method, sample size, analytical method) and applicability (e.g., country of origin, year, sampling stage). For example, polymer-specific information may be critical for systems considering mechanical recycling, as different types of polymers are generally immiscible.^102^ Also, the sampling year and sampling country can be used to assess the uncertainty of the information using data quality matrices.^103^ Furthermore, our database can be used as a starting point for selecting interesting systems to conduct SFAs, e.g. by prioritizing those substances for which a lot of information is available (for a specific region/time frame/product category/polymer), or those that occur in particularly high concentrations, or those that show interesting trends (e.g., decreasing, increasing, recent regulation). Conducting SFAs in the context of mechanical recycling can help to explain current contaminant levels in recycled products and to identify future (cross-)contamination and possible mitigation strategies.^104^

Exposure models help to quantify the dose of a substance that an individual organism or population may be subjected to.^27,28^ Specifically, near-field exposure models require detailed concentration information of a substance in different materials and products. Our LitChemPlast database can help to improve inputs to near-field exposure modeling for plastics by providing accurate real-world concentrations of chemicals in fine-grainend plastic product (sub)categories, and specific regional and temporal resolutions. Substance identities are provided in multiple formats and can be linked to their physicochemical properties (e.g., as model inputs, or to identify substances with similar environmental fate). Substances can also be prioritized based on their exposure potential, which is a combination of their typical concentrations, their release potential from the polymer matrix, and their possible environmental fate. Exposure models and the aforementioned (dynamic) substance flow analysis can also be considered together to predict realistic exposures in various plastic-management scenarios.^105^ Accurate exposure information is critical for understanding which substances pose great risk to the general population or to specific subpopulations (e.g., toddlers or children, people with specific occupations, fence-line communities, or frequent users), and for appropriate risk management (e.g., managing the handling, production, and use of chemicals to create a safer environment for all).

Our LitChemPlast database can also serve as a starting point for: (1) identifying substances of particular interest to science and regulation (e.g., upward trends in the detection frequency or typical concentrations over time, substitutes for legacy substances, substances with similar properties to legacy substances); (2) preliminary screening for “safer” materials that could be preferred stocks for recycling (i.e., product categories that rarely contain known hazardous substances)—such material stock needs to be subjected to thorough testing before recycling, including nontargeted analyses and complementary screening techniques such as bioassays; (3) identifying indicator substances for different polymer types, product categories, or regions that could help in tracing micro- and macroplastic pollution to its origins; and (4) identifying important NIASs and their likely origins by comparison with databases focusing on intentionally added substances (e.g., the PlasticMAP database, Figure 2 – B).

## 5 Implications and Outlook

### 5.1 Implications for Ensuring Safe and Sustainable Recycling

This LitChemPlast database shows that despite considerable efforts by the scientific community to monitor the presence and concentrations of different substances in plastics, substantial gaps remain. It remains difficult to guarantee that recyclates will not be contaminated, in addition to other limitations to the transition of plastics to a safe and sustainable circular economy.^62^ Without prior information, end-of-life plastics containing hazardous substances cannot be recognized and removed before recycling as required to create and maintain “clean cycles”.^40^ The effects of such mismanaged recycling can already be observed in the contaminated recycled plastics that have been tested over the years (section 4.1). The management of plastics within a circular economy, which collectively may contain thousands of different chemicals,^10,11,13,106^ requires corresponding strategies for properly handling the chemicals therein, including preventing exposure to hazardous chemicals.^13,106−108^ Such strategies can be roughly grouped as “cleaner production” and “end-of-pipe”,^107^ and may differ for intentionally used substances and NIASs.

For intentionally used substances, producers/compounders should know what substances they are using and in what concentrations, which may enable both “cleaner production” and targeted “end-of-pipe” strategies. For example, such knowledge may inform stricter regulation or harmonization among producers/compounders of what may be used (e.g., positive lists,^109^ substance bans^56^), as well as technological advances or consumer demand.^108^ Transparency about the chemical compositions of plastics along the supply chain is a prerequisite to support consumer choices and “end-of-pipe” screening and removal of problematic substances. It can be achieved by including them in a digital product passport.^110^ However, this is not without challenges, as information will only be available for new products, may not always be accessible at end-of-life, or different information systems may not be compatible.^111,112^ “End-of-pipe” solutions could include screening and direct removal of hazardous substances. Any information from the producer to the waste treatment facility can support these processes, by targeting the methods to the common problematic substances, or by directly removing common problematic products from the waste streams.

NIASs are more difficult to manage because a variety of factors can influence their presence and concentrations throughout the plastic life cycle.^100^ Some NIASs can be tackled by the plastic producer/compounders by changing processing parameters, e.g., by using “cleaner” raw materials, by using lower temperatures to avoid the production of side or degradation products, or by incorporating additional postproduction cleanup steps (e.g., residual monomer or volatiles removal after polymerization^113,114^). Others may need to be addressed primarily by other actors along the value chains, e.g. avoiding contamination with pesticides and other ubiquitous pollutants during the use phase, or avoiding the exposure of the material to harsh conditions.

In any case, traditional chemical screening of plastic products may not be sufficient for safeguarding a safe and sustainable circular economy, mainly due to the wide range of existing products and as screening will likely have to deal with up to thousands of chemicals per product. This may require a clear screening strategy, for example, (1) prioritizing those regranulates that go into more sensitive applications, (2) focusing on getting an overview of the common contaminants in regranulates using nontargeted analytical techniques, (3) developing fast methods for priority contaminants, or (4) using complementary methods such as high-throughput bioassays to check for biological impacts. As a result, there is an urgent need for the advancement of rapid and uncomplicated screening mechanisms, including effect-directed analysis, but also strategies that go beyond “end-of-pipe” solutions. Such strategies could include the reformulation of plastics including the phase-out of hazardous substances and chemical simplification, enhanced transparency regarding the chemicals in use, the development of inert alternative materials, or innovative business models reducing the reliance on single-use plastics.^110^

### 5.2 Limitations of the Database and Future Development Options

In this study, we present the LitChemPlast database of studies on chemicals measured in various plastics. It may be used as a starting point to (1) prioritize and motivate future measurement campaigns, (2) develop policy measures for ensuring clean material cycles, and (3) support SFAs, exposure assessments, and other applications. Meanwhile, the data we collected are still incomplete, and the data quality and data comparability may be limited in some cases (which is not assessed in this study). These areas would be subject to future development.

**Figure 4:**
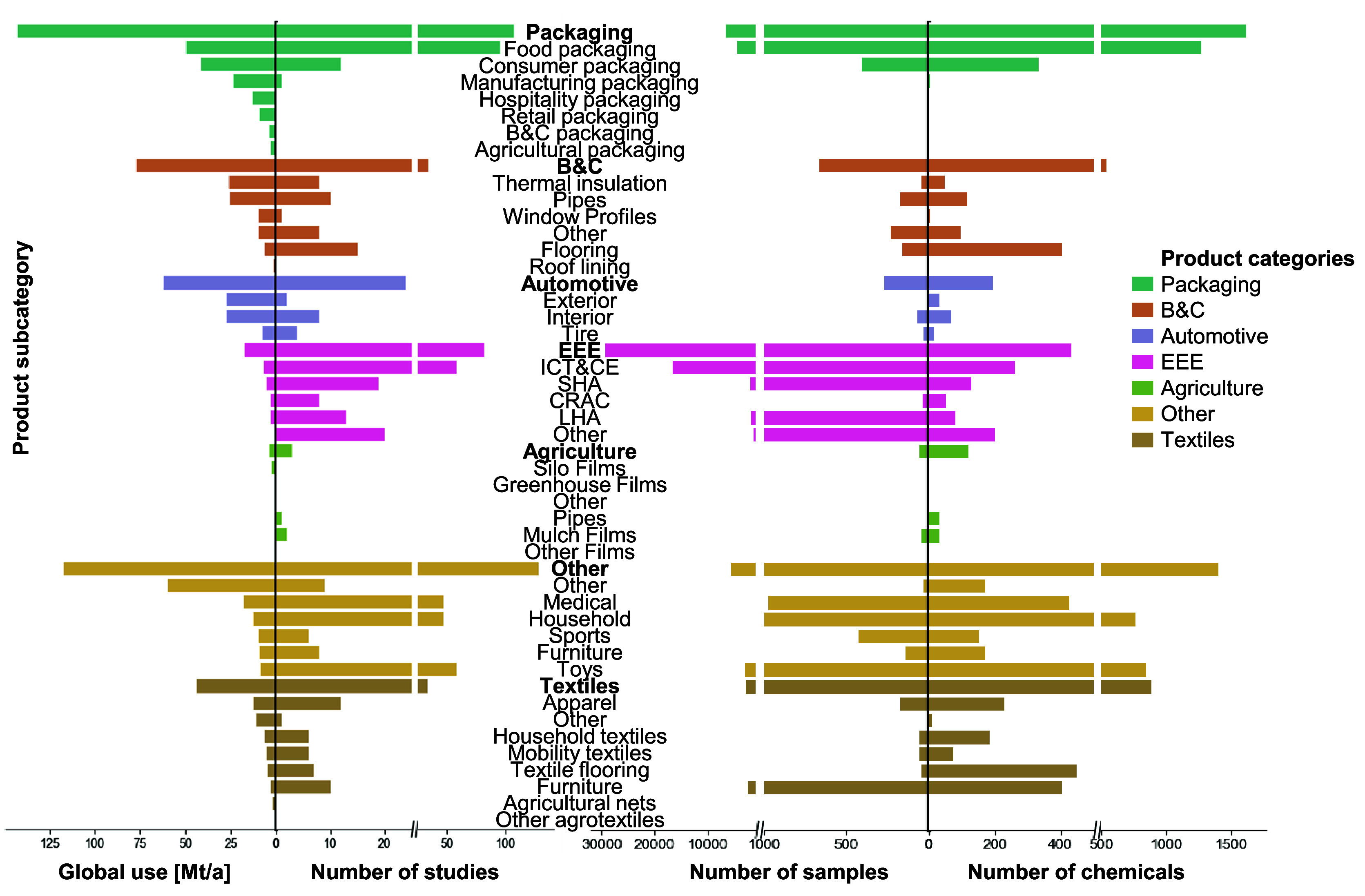
Global use and number of studies, samples, and chemicals by product subcategory. Their global use per subcategory was extrapolated using the global use data by category and the Swiss subcategory use data; see Section S1.4.1 in SI1.^1,32^ The subcategory data may be very similar for other high-income countries, while differences for mid- and low-income countries may be expected.

Some gaps have already been mentioned above (Section 3), i.e., the limited regional coverage with regard to low- and middle-income countries, the lack of nontargeted measurements for product (sub)categories other than (food) packaging, and the narrow focus on well-known hazardous chemicals in targeted measurements. In addition to these general research gaps, certain types of studies may be needed to support the transition to a more circular plastics economy. Several product (sub)categories may be of particular importance for a circular economy, and several types of chemicals need to be monitored to ensure safe use, and potentially safe and sustainable recycling (Table S8 in SI1). For example, comparing the estimated global use amounts of different product subcategories to the number of studies, samples, and measured chemicals reveals that certain subcategories are likely understudied (Figure 4). Particularly, consumer nonfood, manufacturing, and hospitality packaging are rarely studied, despite being among the top users of plastics.

The LitChemPlast database helps to comprehensively understand the presence of chemicals in plastics. Meanwhile, the current database should be seen as a starting point by the scientific and regulatory community. For example, our primary focus on peer-reviewed scientific studies and our literature search may have overlooked other data in the gray literature and may have introduced an unintentional bias toward “positive”, or particularly “interesting”, results due to current publication bias. Future efforts could focus on (I) continuing the literature search to ensure all relevant studies are discovered and included, (II) the systematic incorporation of data on chemicals in textile and food packaging plastics, e.g., by combining this database and other existing efforts (such as the FCCmigex database^45^ or chemicals in textiles databases^48,49^), (III) including important gray literature (such as the regulatory EU SCIP database^42^ or the commercial Pharos database for building materials^115^), and (IV) conducting systematic reviews of the studies, including assessment of the data quality, by following systematic review protocols, such as PRISMA.^116^ Furthermore, advancing publication practices such as by introducing preregistration of planned measurement campaigns, as practiced in other fields such as pharmaceutical or toxicological sciences, could help alleviate publication bias toward positive results.

The categorization of products and chemicals can also be refined, depending on the intended application of the database. For example, exposure assessments might require different “resolutions” regarding product categories than substance flow analyses. The assignment of recycling status, currently based on product reporting/labels, may also be improved in the future, as more advanced approaches, e.g., based on chemical markers or typical fingerprints of recycled plastics, become available.^117,118^

The quality of the presented data varies widely, as different study designs, sampling methods, sample preparation techniques, and analytical methods have been used. Furthermore, many studies did not provide very detailed information or reproducible steps of their sampling procedures, and thus, their results may not provide a good “estimate” of the presence or concentrations of a given substance in the respective product categories and are possibly not reproducible.^119^ As a result, the reported detection frequencies and concentrations are typically not directly comparable. Also, certain uses of the database may require additional data quality assessment by individual users. Future efforts may focus on developing standardized reporting and quality assessments of sampling and analytical methods.

Studies have reported their data in a variety of ways, often ambiguous and not machine-processible, which makes extraction, harmonization, and the reuse of data challenging. For example, many studies have not directly reported their raw data, but only included aggregated results as tables (e.g., median, minimum, maximum) or even just figures showing part of their data. Consequently, the extraction of data required enormous manual work. Furthermore, many studies have reported only (trivial) names of the chemicals, rather than unambiguously specifying the substances they detected by unique identifiers (such as InChIkey, CASRNs). The unique identifiers of these chemicals had to be assigned semiautomatically (using the Python cirpy package) or manually (see Section S1.3.2 in SI1) in this study, which is labor-intensive and error-prone. Future efforts may focus on making published data more “accessible”, e.g., by requiring data reporting to be in line with the FAIR Data Principles.^120^ This might include providing the measurement results for all individual samples in a table, providing unique identifiers for chemicals, and reporting the method LOD/LOQ. Additionally, recent advances in natural language and image processing may help in streamlining and accelerating data extraction. Initial pretests of ChatGPT-assisted data extraction in this study (example in Section S1.3.1 in SI1) showed mixed results, especially when data are “hidden” in graphs or scattered throughout the main paper and the Supporting Information. Machine-extracted results are not included in the current LitChemPlast database. Additional features and improvement of these tools, as well as refined prompts, may achieve more reliable results and could be used in a future development of the database.

The LitChemPlast database is a starting point in providing concentration data for chemicals in plastics. Overall, concerted efforts, including harmonization and enhanced transparency, will be needed to provide a comprehensive understanding of chemicals in plastics, their concentrations, and associated risks to human health, the environment, and the transition to a circular economy. We encourage the scientific and regulatory communities to further develop and use the database.

## Data Availability

The LitChemPlast database (including any updates) is publicly available on Zenodo under: 10.5281/zenodo.13271346

## Supporting Information Available

Supporting Information 1 (SI1): method details (Section S1) and additional results (Section S2) (PDF)
Supporting Information 2 (SI2): the current version of the database created for this publication (LitChemPlast database) (XLSX)
Supporting Information 3 (SI3): the Python script (as Jupyter Notebook) used for data analysis and the creation of the graphics in this manuscript (ZIP)

## Notes

A previous version of this database was used in the PlastChem project.^13^ A previous version of this manuscript was published as Chapter 3 in the doctoral thesis of H.W.^121^
