# Supplementary material for: LitChemPlast: An Open Database of Chemicals Measured in Plastics
Source: Environ Sci Technol Lett. 2024 Oct 29;11(11):1147–60. doi: 10.1021/acs.estlett.4c00355 (PMC11562724; doi:10.1021/acs.estlett.4c00355)
Supplement: Supplementary file 1 — ez4c00355_si_001.pdf [file ez4c00355_si_001.pdf]

Supporting Information: LitChemPlast – an open database of chemicals measured in plastics

Helene Wiesinger,[*,†,‡] Anna Shalin,[†,¶] Xinmei Huang,[†,§] Armin Siegrist,[†,‖] Nils Plinke,[†] Stefanie Hellweg,[†,‡] and Zhanyun Wang[*,†,‡,⊥]

†*Chair of Ecological Systems Design, Institute of Environmental Engineering, ETH Zürich, 8093 Zürich, Switzerland*

‡*National Centre of Competence in Research (NCCR) Catalysis, Institute of Environmental Engineering, ETH Zürich, 8093 Zürich, Switzerland*

¶*University of Toronto, Toronto, ON M5S 1A1, Canada*

§*Department of Environmental and Occupational Health Sciences, University of Washington, Seattle, WA 98195, United States*

‖*Laboratory of Sustainable Food Processing, Institute of Food, Nutrition and Health, ETH Zürich, 8092 Zürich, Switzerland*

⊥*Empa - Swiss Federal Laboratories for Materials Science and Technology, Technology and Society Laboratory, 9014 St. Gallen, Switzerland*

E-mail: wiesinger@ifu.baug.ethz.ch; zhanyun.wang@empa.ch

Submitted to: *Environmental Science & Technology Letters.*

Manuscript ID: ez-2024-00355h.R1

Date of submission: August 22nd 2024

Supporting Information - File 1 (SI1)

Contents

Supporting Information - File 1 (SI1) **S2**

List of Figures **S3**

List of Tables **S5**

S1 Detailed methods **S6**
- S1.1 Overview . . . S6
- S1.2 Data sources . . . S8
- S1.3 Data extraction and processing . . . S11
  - S1.3.1 Extraction of information from the literature . . . S11
  - S1.3.2 Data harmonization . . . S13
- S1.4 Data analysis . . . S20
  - S1.4.1 Inclusion of further information . . . S20

S2 Additional results **S22**
- S2.1 Overview of the data . . . S22
  - S2.1.1 Regional coverage . . . S22
  - S2.1.2 Samples . . . S24
  - S2.1.3 Reported Substances . . . S25
- S2.2 Identification of gaps and research needs . . . S35
  - S2.2.1 Recyclability . . . S36
  - S2.2.2 Exposure potential . . . S39
- S2.3 Potential effects of mechanical recycling . . . S40
  - S2.3.1 Time-series of polybrominated diphenyl ether (PBDE) concentrations S40

References **S47**

List of Figures

S1 Focus of the LitChemPlast database compared to the PlasticMAP database S6
S2 Simplified database diagram of the LitChemPlast database. S7
S3 Overview of the extracted literature over time. S9
S4 Wordclouds for title and topics or keywords of the studies reviewed. S10
S5 Regional coverage as maps by the number of studies (left) and the number of samples (right). S22
S6 Regional coverage as bar plots by the number of studies (left) and the number of samples (right). S23
S7 Number of the targeted and non-targeted studies conducted per substance type and product (sub-)category, sampling stage, and polymer type. S24
S8 Details of the Venn diagrams depicted in Figure 2 – B in the manuscript. . . S25
S9 Major substances by the number of studies. S26
S10 Top 73 substances covering 25% of all the sample-study combinations. S28
S11 Top 73 non-targeted substances by the sample-study combinations. S29
S12 Top 20 targeted substances regarding the number of studies, the tested samples, their detection frequency (DF) and the measured concentrations. S30
S13 Major substances by the number of samples. S31
S14 Top 45 substances covering 50% of all the sample-substance combinations. . S32
S15 Top 45 non-targeted substances by the sample-substance combinations. . . . S33
S16 Number of chemicals and samples tested by study. S37
S17 Global use and number of studies, samples, and chemicals by product subcategory. S38
S18 Number of studies (left) and samples (right) by the product subcategory, compared to their exposure potential. S39
S19 Concentrations of all measured PBDEs over time, distinguished by recycling status. S40
S20 Concentrations of measured polybromodiphenyl ethers, that could not be assigned to any other subgroup, over time distinguished by recycling status. . . S40
S21 Concentrations of decabromodiphenyl ether (CASRN:1163-19-5) over time, distinguished by recycling status. S41
S22 Concentrations of all measured nonabromodiphenyl ethers over time, distinguished by recycling status. S41
S23 Concentrations of all measured octabromodiphenyl ethers over time, distinguished by recycling status. S42
S24 Concentrations of all measured heptabromodiphenyl ethers over time, distinguished by recycling status. S42

S25 Concentrations of all measured hexabromodiphenyl ethers over time, distinguished by recycling status. S43
S26 Concentrations of all measured pentabromodiphenyl ethers over time, distinguished by recycling status. S44
S27 Concentrations of all measured tetrabromodiphenyl ethers over time, distinguished by recycling status. S45
S28 Concentrations of all measured tribromodiphenyl ethers over time, distinguished by recycling status. S45
S29 Concentrations of all measured dibromodiphenyl ethers over time, distinguished by recycling status. S46

List of Tables

S1 Hierarchical product categories assigned to the different samples from the literature. S16
S2 Polymer types assigned to the different samples from the literature S18
S3 Sampling stage assigned to the different samples from the literature S19
S4 Sampling procedure assigned to the different methods used in the literature . S19
S5 Sources of the use amounts of different plastic product (sub-)categories. . . . S21
S6 Major substances according to the number of studies (n) by type of analysis (targeted vs non-targeted), as displayed in Figure 2 – A in the main manuscript. S27
S7 Overview of available data for different (sub-)product categories, as displayed in Figure 4 in the main manuscript. S35
S8 Factors for recycling-relevant case studies. S36

S1 Detailed methods

S1.1 Overview

To provide an overview of substances currently present in plastics and assess future research needs, we extracted the measurement results from previously conducted measurement campaigns of chemicals in plastics. Our previous database, the PlasticMAP database,[S1] has focused on substances used in the production of plastics. In comparison, this database, the LitChemPlast database, focuses on the chemical composition of real-world plastics, based on measurement campaigns (Figure S1).

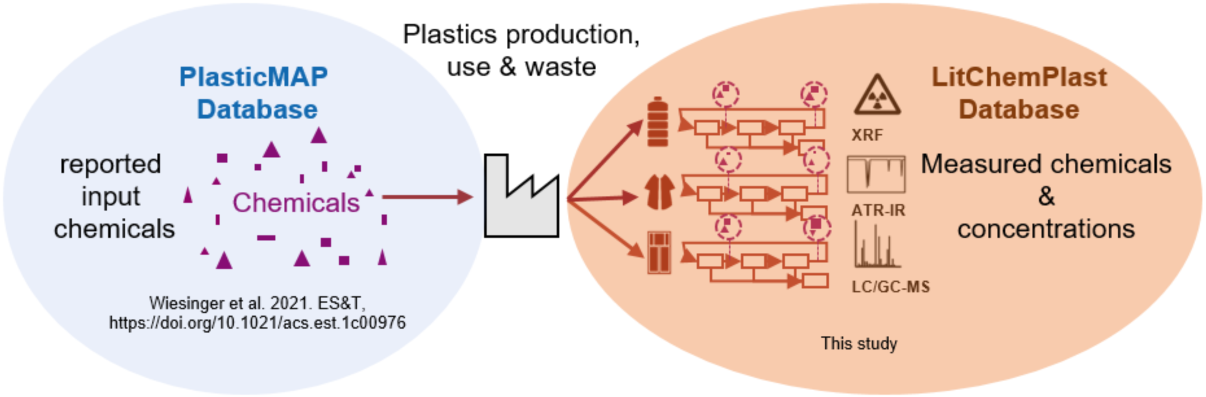

Figure S1: Focus of the LitChemPlast database compared to the PlasticMAP database[S1]

The database is presented in full in the Supporting Information 2, SI 2, as an Excel file. The current version and all updates will be available on Zenodo (`https://doi.org/10.5281/zenodo.13271346`). To ensure the ease of use, we have provided the data in two formats (Figure S2): (1) a “Mastertable” (Sheet “LitChemPlast”) and (2) a simple relational database (connecting the Sheets “Literature”, “LiteratureChemicals”, “Chemicals”, “Sample” in the depicted manner).

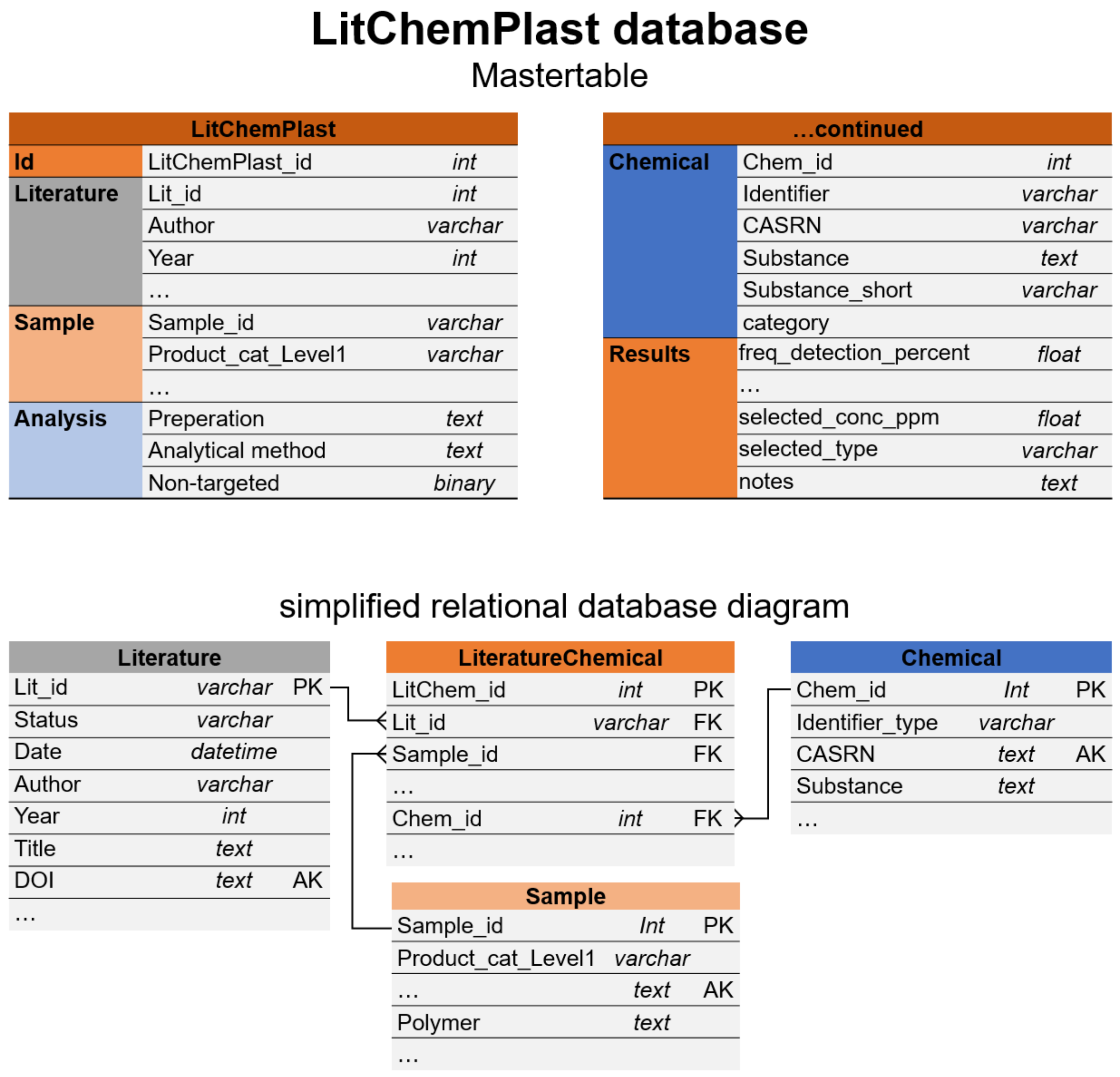

Figure S2: Simplified database diagram of the LitChemPlast database. The three main tables and their links are shown. Only one-to-many links are relevant for the LitChemPlast database. PK = primary key (id, that uniquely identifies each row in a table), AK = alternative key ("natural unique identifier"), FK = foreign key (matches the primary key column of another table, relating the tables to one-another).

S1.2 Data sources

To ensure a good overview, both database search (of *Web of Science* and *SciFinder*) and manual search of related studies (*citing* and *cited by*) were conducted. Studies were considered relevant if they reported the measured chemical composition of plastics. Plastics of all life-cycle stages (raw material, market, use, waste) and product categories were included. Studies conducted by industry, government or civil society organizations were included, even when they were not published in scientific journals. Studies for which no full text could be retrieved were excluded.

The identification of relevant studies began with searches and direct screening of results on Web of Science and SciFinder, using keywords containing at least one keyword related to plastics (i.e., "plastic", specific plastic product categories, and/or polymer types) and another one related to measured chemicals (i.e., "chemical", and/or specific analytical methods such as "XRF," "GC-MS," or "LC-MS"). The specific plastic product categories in Table S1 (Category - Level 1, and for "Others", also Subcategory - Level 2) and polymer types in Table S22 (up to resin code 6) were used as the keywords. "Food packaging" and "Textiles" were not used as keywords, for the reasons explained in the main text.

The results were sorted by "relevance" using the databases' own algorithms,[S2] and the top results were screened for relevance based on their titles. Those that appeared relevant were downloaded for the subsequent full-text screening. We did not document the total number of studies reviewed by title directly screened on Web of Science and SciFinder.

During the full-text screening, if a study was confirmed as relevant (reporting the measured chemical composition of plastics), related studies (either *citing* or *cited by* the relevant study) were also downloaded and reviewed if their titles indicated potential relevance. This method ensured a broader capture of significant research.

Overall, the search and selection process resulted in reviewing the full texts of 786 studies. From these, 372 studies were identified as relevant, and their data were extracted. Studies mostly appeared in the journals Environmental Science & Technology (15 %), Science of the

Total Environment (10 %), and Food additives and contaminants (5 %). More than 60 % were published after 2010 (Figure S3).

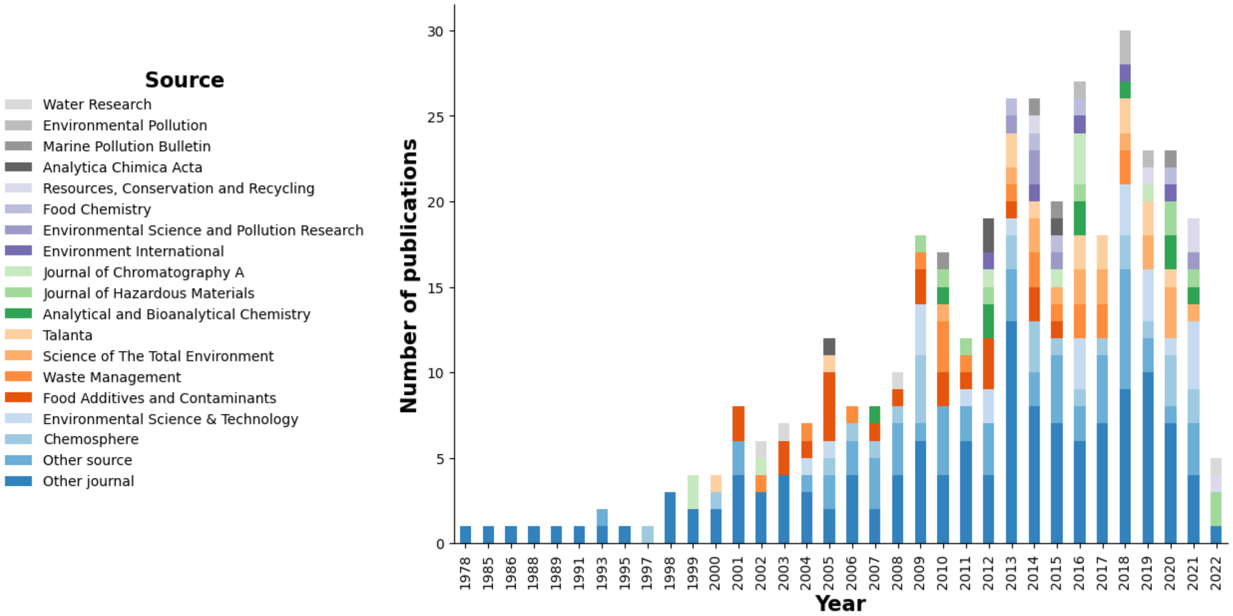

Figure S3: Overview of the extracted literature over time. Other journals include Environmental Science: Processes and Impacts, Molecules, International Journal of Pharmaceutics, Polymer Testing, Food and Chemical Toxicology, Journal of Chromatography B, Analytical Methods, Journal of Agricultural and Food Chemistry, or Indoor Air. Other sources mainly include governmental reports, civil society organization reports, non-peer-reviewed scientific reports, or laboratory equipment protocols.

The most common words appearing in the titles and topics of the considered studies are "plastic", "migration", "waste", "food packaging", "flame retardant", and "GC-MS" (Figure S4). The common appearance of the terms "migration" and "food packaging" is surprising, considering that no specific efforts were made to identify studies related to food packaging, as great resources, such as the FCCMigex database,[S3] already exist.

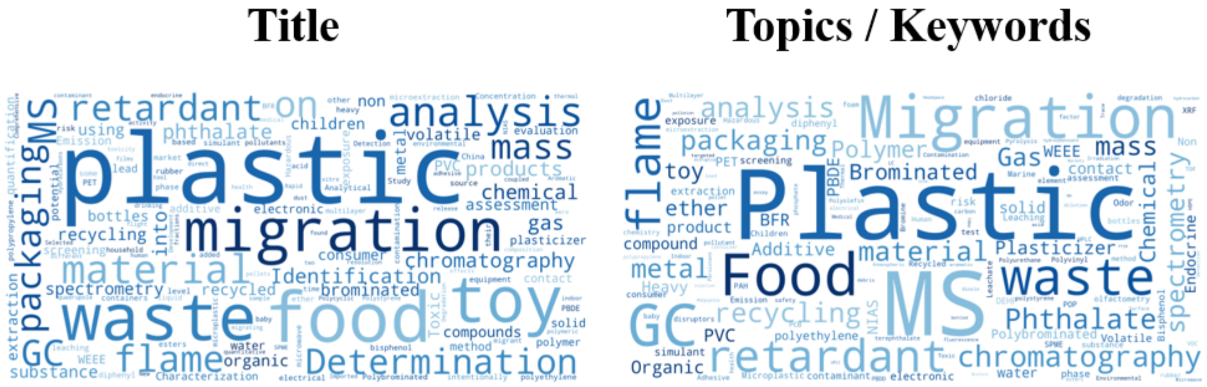

Figure S4: Wordclouds for title (right) and topics or keywords (left) of the studies reviewed. Common words (e.g. "a", "the", "and") were excluded from the analysis.

S1.3 Data extraction and processing

S1.3.1 Extraction of information from the literature

For each study, the following types of information were manually extracted where possible: (1) bibliographic information including author names, journal name, publication year, abstract and keywords, (2) information on the examined samples, including their product categories (Table S1), their polymer types (Table S2), their sampling stage (Table S3), and their recycling status, (3) information on the sampling, including sample size, country, year, and selection procedure (Table S4), (4) the sample preparation and analytical method, (5) information on the examined chemicals (Section S1.3.2) and (6) the determined detection frequencies and concentrations in mg/kg for each sample–chemical combination.

Information was manually extracted from each study to ensure relevance and improve data quality (see Section S1.3.1). In total, five people were involved in the data extraction (namely: the co-authors HW, ASh, XH, NP, and ASi). Training included extracting studies whose data had been extracted before by HW, discussing the results, and resolving any discrepancies. When it was unclear which data in a study are relevant, these situations were discussed and resolved with HW.

Typically, the determined concentrations were found in the results section in the main text, tables or graphs, or in the supporting information. Ideally, the authors reported information for individual samples, otherwise the reported information for several samples was extracted noting down the sample size. Where necessary, concentration units were converted during the data extraction to $mg_{substance}/kg_{plastic}$ in the following manner:

(1) concentrations in mass/mass (substance/plastic): simple conversion (e.g., $1\,mg\,g^{-1} = 1\,000\,mg\,kg^{-1}$)

(2) concentrations in mass/Volume (substance/plastic): conversion by average density of the material (e.g. HDPE: $0.95\,kg\,L^{-1}$)

(3) concentrations in solvent: checking how much plastic material was dissolved in the

solvent in the first place and back-calculating to the substance/plastic concentration (e.g., result concentration 1 µg mL^{-1} (substance/solvent) divided by 1 mg mL^{-1} (plastic/solvent) = 1 µg mg^{-1} (substance/plastic) = 1 000 mg kg^{-1})

(4) "Area" or "Signal": concentration was not reported, and thus, was not included

(5) Other reports: if a conversion to mass/mass (substance/plastic) was possible but did not strictly follow the above examples, the conversion method was described in the comments for that data row.

All other information was typically found in the methods in the main text of the study. Some sources did not contain the required information or were ambiguous in their reporting. In particular, information on the recycling status, the sampling year and the sampling procedure was often lacking. The following types of information could be (at least partially) extracted for the original studies: product category - 331 studies (89 % of all 372 studies; generic terms, such as "waste", "plastic", "litter", or "granules", were counted as no information here), polymer type - 259 studies (70 %), recycling status - 42 studies (11 %), sampling country - 288 studies (77 %), sampling year - 110 studies (30 %), sampling procedure - 235 studies (63 %; of these 103 studies performed "grab sampling", i.e., they followed no procedure to ensure representativeness). Note, that several studies mentioned relevant information for some samples but not for others.

Extraction pre-tests employing ChatGPT The ability of ChatGPT to support the data extraction process in the future was tested with one study.[S4] The chat with and output by ChatGPT can be found here:

`https://chat.openai.com/share/2b093249-4685-472d-836d-4563192781ed`

While ChatGPT was able to extract some data from the article correctly and provided them in the desired format after revising the prompts several times, it did not extract all the data contained in the article. Specifically, more substances were targeted, but only two were found by ChatGPT. This means the information about the other substances not being

present in the tested samples (i.e. detection frequency of 0 %) was not correctly extracted.

Thus, ChatGPT is likely not yet able to replace manual extraction of new studies, and could not be used in this study for data extraction. Nevertheless, further testing more than one study and improving the prompts, is required to assess its overall performance. Improved versions of ChatGPT or other specialized machine-learning data extraction tools may enable human-machine collaboration for data extraction in the future.

S1.3.2 Data harmonization

The diverse ways in which researchers reported their data made harmonization necessary. The main goals were

(1) to assign each reported chemical its standard CASRN, or assign it to a chemical type without CASRNs (i.e. "group of substances", "element"),

(2) to assign each sample a product (sub-)category, polymer type, and recycling status

(3) to assign each entry the sampling procedure, sample size, and analytical method. These harmonized data allow, to a certain extent, for assessing the data quality.

Chemicals The aim was to harmonize the provided chemical information (commonly trivial names), by linking them to their CASRNs. The following procedure was followed:

- Groups (e.g. "total brominated flame retardants", "poly-aromatic hydrocarbons") are not represented by single CASRNs, and thus, were not treated further.

- Elements (e.g. "Pb", "iron") are not represented by single CASRNs, but can rather be linked to substances containing them. The intentionally added substances from the PlasticMAP database containing the found Elements were identified (Figure 2 – B in the main text)

- Names and acronyms were matched to their CASRN wherever possible:

1. All entries were automatically searched using cirpy (`https://cirpy.readthedocs.io/en/latest/`, see the python script in SI3) and the Comptox batch search (`https://comptox.epa.gov/dashboard/batch-search`).
2. Missing entries were manually searched by using SciFinder (`https://scifinder.cas.org/`) and/or PubChem (`https://pubchem.ncbi.nlm.nih.gov/`).
3. Missing entries were manually searched on other websites (i.e. Google, Chemicalbook, Wikipedia).
4. Remaining entries were considered "not assignable"

Identified CASRNs were finally checked for registration at SciFinder (`https://scifinder.cas.org/foralternative/oldCASRNs`) and by using the check-digit method (`https://www.cas.org/support/documentation/chemical-substances/checkdig`). Leading non-numeric characters or leading zeros present in some CASRNs were removed.

- Relevant names, identifiers, and other substance information was retrieved from Comptox batch search (`https://comptox.epa.gov/dashboard/batch-search`)

The identified substances were assigned to "groups" based on the most commonly targeted substances (e.g. "*ortho*-phthalates", "metals", "BFRs", "PFRs"). The assignment was based on structural features, identified partially by the molecular formula, the name, or the SMILES. All assignments were manually double-checked.

Product categories, polymer types and sampling The samples described in the literature were assigned to harmonized polymer types (Table S2), product categories on different hierarchical levels of detail (Table S1), and life-cycle / sampling stages (Table S3). The employed categories were based on the recent material flow analyses of plastics.[S5,S6] The original literature did not always contain an appropriate level of detail for indisputable assignment. In some cases, no information or insufficient information on the exact sample was provided (e.g. "bottle" may be assigned to "Packaging" *(id=1_0)*, but could also be part

of “Household items” *(id=3_5)* if it is a “reusable bottle”). In other cases, especially when waste was sampled, the sample may have been a mixture of different product categories. Generally, the most likely assignment was made; however, if the description was ambiguous, “unknown”/“not specified” was assigned.

Polymer types were harmonized to individual polymer types (Table S2). Polymer types (and other materials) in multimaterials were separated by “/”.

The sampling method was characterized by the sampling stage, the sampling country, the sampling year, the sampling size, and the sampling procedure. The sampling stage is which “life-cycle-stage” the sampled plastics were in, and was assigned based on the categories in Table S3. The sampling procedure explains what was done to ensure representative sampling and was assigned using the categories in Table S4. The extracted years were checked for plausibility: they had to be before the study publication, and they had to be an integer.

Table S1: Hierarchical product categories assigned to the different samples from the literature. Cat. = Category, B&C = Building & construction, Auto. = Automotive, EEE = Electrical and Electronic Equipment, PTT = Pots, tubs, and trays, PET = polyethylene terephthalate, EPS = expanded polystyrene, PVC = polyvinyl chloride.

**ID** Level	**Cat.** 1	**Subcategory** 2	**Group** 3	**Description or examples**
0	Generic	-	-	All samples, without specific description
0_r	Generic	Raw material	-	Granulates, flakes, masterbatches without specific information on their use
0_w	Generic	Waste	-	Waste without specific origin (e.g. municipal solid waste)
1	Packaging	Generic	-	-
1_1	Packaging	Food packaging	Generic	Food packaging used by final consumer
1_1_1			Films	e.g. candy wrappers
1_1_2			Bags	e.g. chips bags
1_1_3			Bottles	e.g. water PET bottles
1_1_4			PTTs	e.g. yoghurt cups
1_1_5			Other	e.g. mayonnaise tubes
1_2	Packaging	Consumer packaging	Generic	Non-food packaging used by final consumer
1_2_1			Films	e.g. cling films around EEE or clothing
1_2_2			Bags	e.g. shopping bags
1_2_3			Bottles	e.g. detergent bottles
1_2_4			PTTs	e.g. trays for cosmetics
1_2_5			Other	e.g. EPS beads for transport
1_3	Packaging	Manufacturing packaging	Generic	Packaging used at manufacturing sites
1_3_1			Films	e.g. shrink film for pallets
1_3_2			Rigids	e.g. boxes and bottles at manufacturing sites
1_4	Packaging	Retail packaging	Generic	Packaging used at retailers, not at final consumers
1_4_1			Films	e.g. shrink films and bags for secondary packaging in back of store
1_4_2			Other	e.g. boxes and other rigids in back of store
1_5	Packaging	Hospitality packaging	Generic	Food and non-food packaging used in hospitality industry
1_5_1			Films	e.g. restaurant-size bags and cling films
1_5_2			Bottles	e.g. large bottles for sauces or oil
1_5_3			PTTs	e.g. large bucket for sauces
1_5_4			Other	e.g. tubes and specialized food-packaging
1_6	Packaging	B&C packaging	Generic	Packaging used at building sites
1_6_1			Films	e.g. shrink film around bricks
1_6_2			Rigids	e.g. transport buckets, boxes
1_7	Packaging	Agricultural packaging	Generic	Packaging used for agriculture
1_7_1			Films	e.g. bags for fertilizer or seeds
1_7_2			Rigids	e.g. pesticides bottles

ID<br>Level	Cat.<br>1	Subcategory<br>2	Group<br>3	Description or examples
2	B&C	Generic	-	-
2_1		Pipes and ducts	-	e.g. water pipes
2_2		Thermal insulation	-	e.g. EPS foam for insulation
2_3		Flooring	-	e.g. Vinyl indoor flooring
2_4		Window profiles	-	e.g. PVC window profiles
2_5		Roof lining	-	e.g. layer beneath roof tiles
2_6		Other B&C products	-	e.g. plastic tiles and plates
3	Auto.	Generic	-	-
3_1		Exterior	-	e.g. bumper
3_2		Interior	-	e.g. instrument panel
3_3		Tire	-	-
4	EEE	Generic	-	-
4_1		Large household appliances (LHA)	-	e.g. stoves, ovens, dishwashers
4_2		Cooling, refrigerating and air conditioning devices (CRAC)	-	e.g. fridges, freezers, air-conditioning
4_3		Small household appliances (SHA)	-	e.g. hairdryers, toasters, vacuums
4_4		ICT equipment and consumer electronics (ICT&CE)	-	e.g. phones, computers, printers, audio and video equipment
4_5		Other EEE	-	e.g. EE toys, sports items, tools
5	Agriculture	Generic	-	-
5_1		Silo Films	-	-
5_2		Greenhouse Films	-	-
5_3		Mulch Films	-	-
5_4		Other Films	-	e.g. insulation films
5_5		Pipes	-	e.g. irrigation pipes
5_6		Other agricultural products	-	e.g. hooks, brackets, clips
6	Others	Generic	-	-
6_1		Household items	-	e.g. storage boxes, reusable drinking bottles, laundry baskets
6_2		Toys	-	e.g. Rubick's cube
6_3		Furniture	-	e.g. chairs, tables
6_4		Sports items	-	e.g. balls, rackets
6_5		Medical and hygiene items	-	e.g. syringes, blood bags
6_6		Other products	-	Any remaining products, e.g. traffic cones
7	Textiles	Generic	-	-
7_1		Apparel	-	e.g. T-shirts, socks
7_2		Household textiles	-	e.g. bedlinen, blankets, curtains
7_3		Textile flooring	-	e.g. built-in carpet floor, carpets
7_4		Textile furniture	-	e.g. couches, chairs etc. with textile covers
7_5		Mobility textiles	-	e.g. car carpets, seat covers, air filter
7_6		Agricultural nets	-	e.g. insect nets, anti-hail nets
7_7		Other agrotextiles	-	e.g. bands, non-woven fabric
7_8		Other textiles	-	e.g. geotextiles, building textiles, EEE filters

Table S2: Polymer types assigned to the different samples from the literature

Polymer type	Polymer name	Resin code
PET	polyethylene terephthalate	1
HDPE	high density polyethylene	2
PVC	polyvinyl chloride	3
LDPE	low density polyethylene	4
PP	polypropylene	5
PS	polystyrene	6
EPS	expanded polystyrene	6
ABS	acrylonitrile butadiene styrene	7
HIPS	high impact polystyrene	7
PA	polyamides	7
PC	polycarbonate	7
PUR	polyurethanes	7
Others	e.g. Cellophane, polybutylene terephthalate (PBT), polyhydroxyalkanoate (PHA), Ethylene-vinyl acetate (EVA), Ethylene-vinyl alcohol (EVOH)	7
Multilayer	*polymers or materials were separated by "/"*	
Not specified	-	-

Table S3: Sampling stage assigned to the different samples from the literature

**Sampling stage**	**Description**	**Examples**	**Considerations**
Raw Material	Samples of granulate before the product manufacturing	granulates, flakes, master batches	- Known production time (now)<br>- Homogeneous samples<br>- Unknown product category
Market	Samples bought from retail places	newly bought products, products in stores	- Known production time (recently)<br>- Heterogeneous samples<br>- Clear product category
Use	Samples in the use phase	products in a household, products consumed by industry	- Varied and unknown production time (within product lifetime)<br>- Heterogeneous samples<br>- Clear product category
Waste	Samples / batches of discarded products	waste collectors, waste treatment	- Varied and unknown production time (around end of life-time)<br>- Very heterogeneous samples<br>- Less clear product category
Waste - Regranulate	Samples of granulate after recycling	Reprocessed waste of a specific product group	- Varied and unknown production time (around end of life-time)<br>- Heterogeneous samples<br>- Less clear product category

Table S4: Sampling procedure assigned to the different methods used in the literature

**Sampling procedure**	**Description**	**Examples**
Random	Draw randomly from all available samples	n.a. (idealized sampling, not possible in reality)
Systematic	Collect every n^{th} sample	select from conveyor belt
Stratified	Collect from discrete population segments or product groups	select representative samples from each product groups (e.g. by market relevance)
Cluster	Collect from existing subgroups of the entire population	select all samples from one store, one household,...
Grab sampling	Collect what you find first without regard for representativeness	any plastic bottle discarded in front of the lab

S1.4 Data analysis

All data analysis was conducted using the python scripts included in Supporting Information 3 (SI3).

S1.4.1 Inclusion of further information

Other information, such as the hazard data (Section S1.4.1), the substances used in the production of plastics - the PlasticMAP database[S1] (Section S1.4.1), and the use amounts in metric tonnes of different plastic products (Section S1.4.1) were included from further data sources.

GHS Data - eChem Portal Hazard data concerning human toxicity and ecotoxicity (e.g. CMR, AqTox, STOT-RE, Sens) were retrieved from the eChemPortal by the Organisation for Economic Co-operation and Development (OECD). The portal contains harmonized hazard classifications from national institutions, in line with the globally harmonized system (GHS). Under "classification search" each endpoint and its subcategories was searched and downloaded as an Excel file that contained all substances fulfilling this endpoint. All excel files were combined and the entries in the "Classification" columns were used for the final hazard data assignment. A table with all relevant GHS Hazard Codes for all substances was created.

PlasticMAP database The comparison to the PlasticMAP database[S1] was done via Python. Both databases use CASRNs as the identifier for individual substances. However, some CASRNs link several substances (i.e. deleted or alternative CASRNs). To ensure that all overlapping substances are recognized, all relevant CASRNs of substances in PlasticMAP were compared with the ones recorded in LitChemPlast.

The overlap regarding elements was based on the "Substance" grouping in PlasticMAP. Essentially the present elements for all substances in PlasticMAP are known, and thus, all

substances containing an element detected in a screening study may be identified.

Use amounts for plastic subcategories The use amount of plastics for different product categories (M_{cat}) and subcategories(M_{sub}) were retrieved from different sources (Table S5). For the comparison of the number of studies to the total production volume, the subcategories from the recent study by Klotz et al. (2022) were used as they are fine-grained.[S6] Use amounts in this granularity were not available on a global level and thus extrapolated from the Swiss subcategory fractions and the global category flows (based on equation S1).

$$M_{sub,global} = M_{cat,global} * \frac{M_{sub,CH}}{M_{cat,CH}} \tag{S1}$$

Table S5: Sources of the use amounts of different plastic product (sub-)categories. OECD = Organisation for Economic Co-operation and Development.

**Source**	**Scope** *Regional*	*Temporal*	*Available product levels*
OECD[S7]	Global	2019	Categories ($M_{cat,global}$)
Klotz et al. (2022)[S6]	Switzerland	2017	Categories ($M_{cat,CH}$) & Subcategories ($M_{sub,CH}$)

S2 Additional results

S2.1 Overview of the data

S2.1.1 Regional coverage

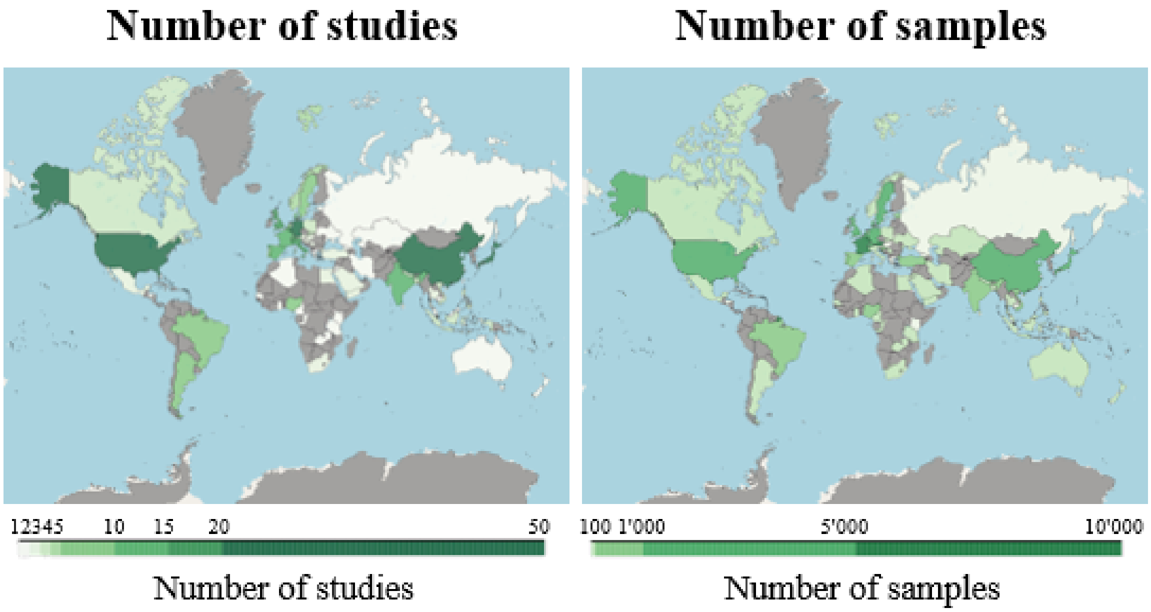

Figure S5: Regional coverage as maps by the number of studies (left) and the number of samples (right).

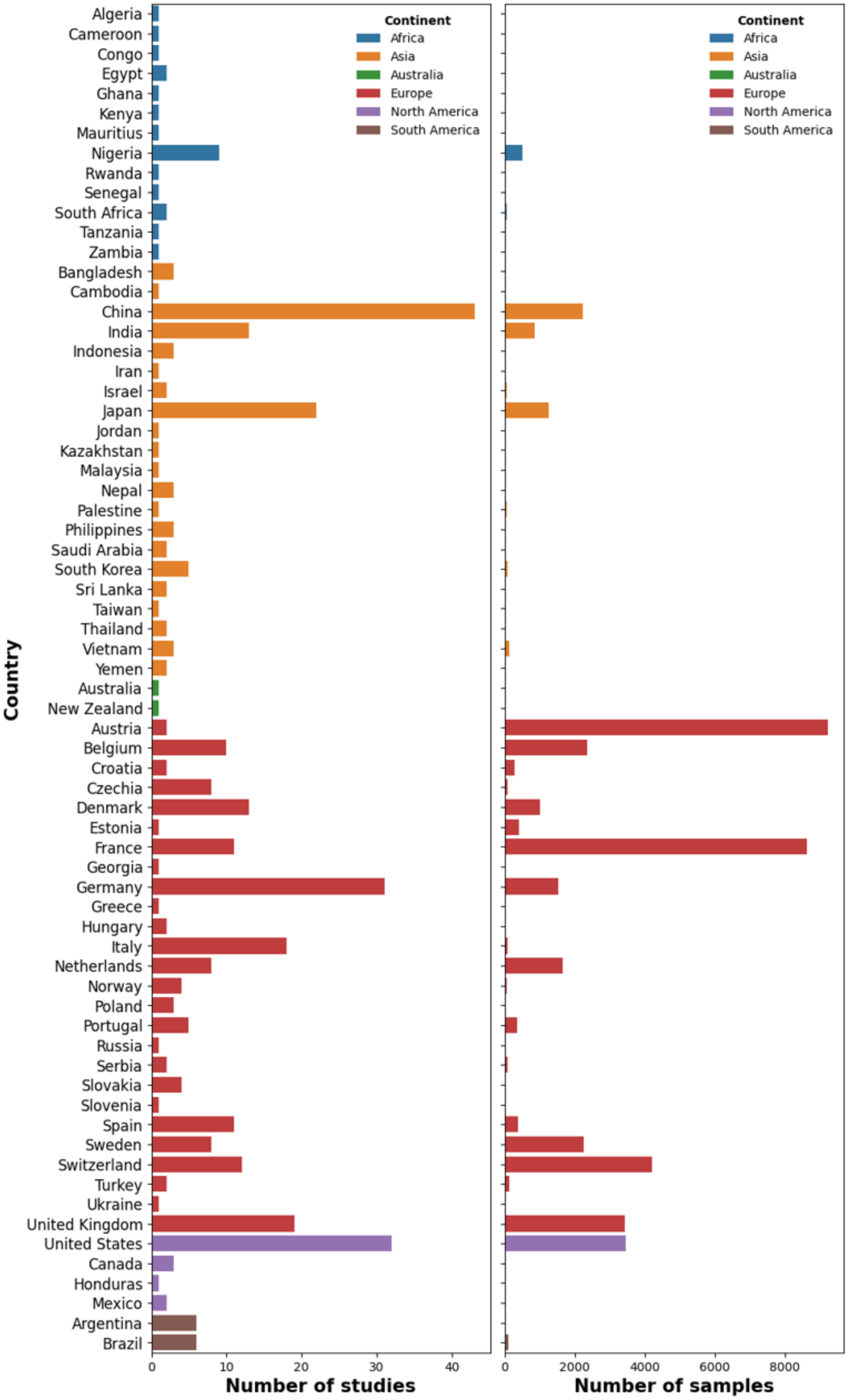

Figure S6: Regional coverage as bar plots by the number of studies (left) and the number of samples (right).

S2.1.2 Samples

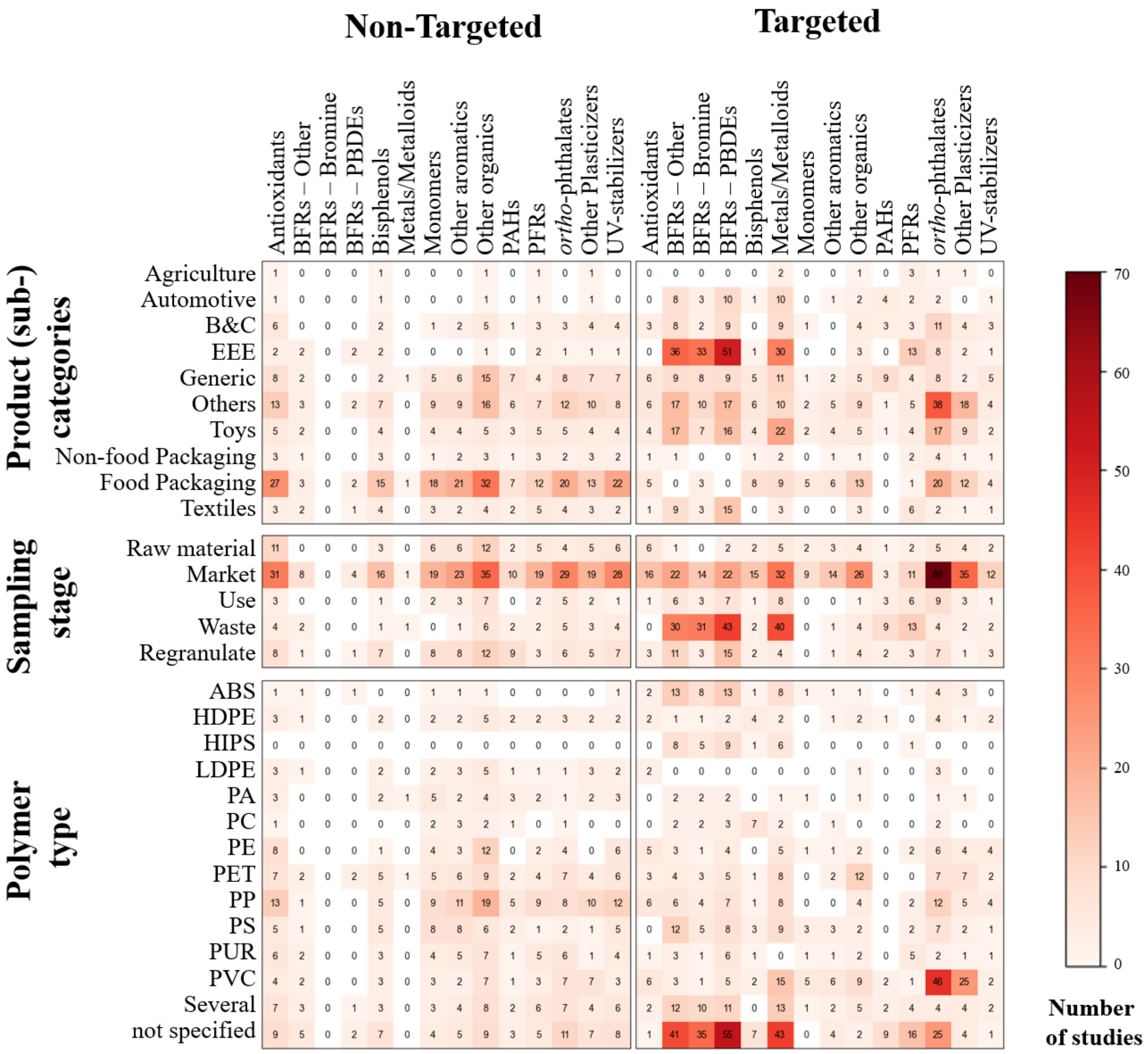

Figure S7: Number of the targeted and non-targeted studies conducted per substance type (top) and product (sub-)category, sampling stage, and polymer type (side). The exact number of studies is written to ease data extraction from heatmaps. The product (sub-)category and sampling stage are shown without numbers in the main manuscript - Figure 1

S2.1.3 Reported Substances

The overlap of the LitChemPlast database with the PlasticMAP database[S1] is depicted in the main part of the manuscript (Figure 2 – B). However, the size of each overlap was not shown, but can be found in Figure S8.

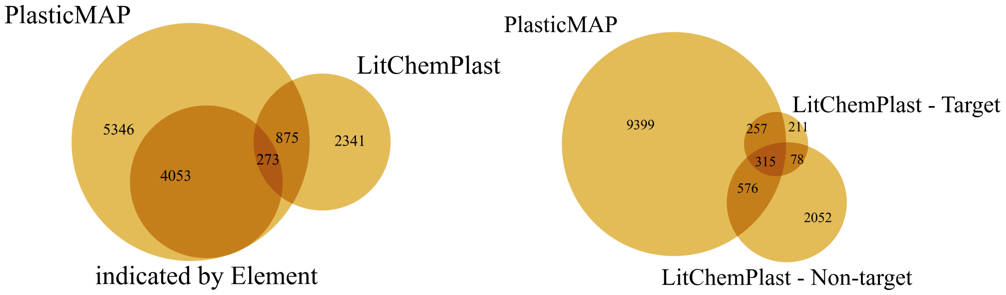

Figure S8: Details of the Venn diagrams depicted in Figure 2 – B in the manuscript. PlasticMAP is a database of intentionally used monomers, additives, and processing aids.[S1]

The "most important" / "major" substances can be determined in various ways. For example, by looking at the number of studies or the number of samples, in which they were targeted or found (in case of non-targeted approaches). In the main text, only the number of studies was displayed, here additional graphics and information can be found.

Number of studies The number of studies in which a substance was targeted or detected varied widely (Figure S9). The major substances (top 20) that were depicted in Figure 2 – A are shown in Table S6.

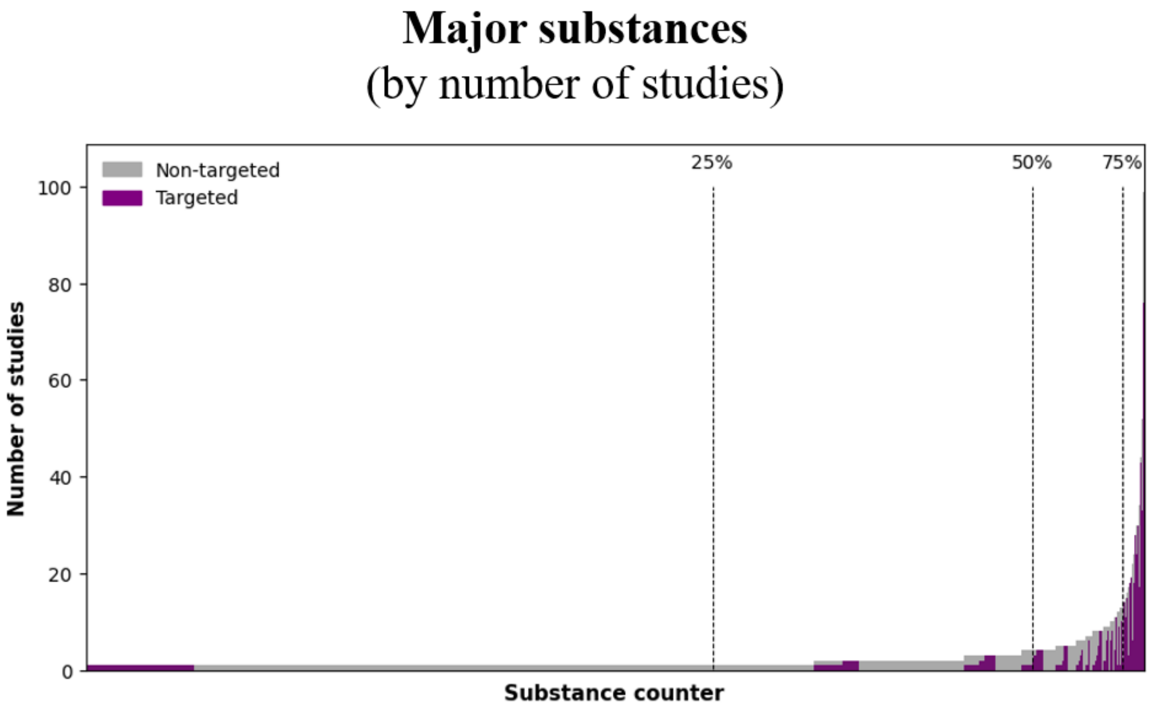

Figure S9: Major substances by the number of studies. 75% of study-substance combinations are covered by the top 137 substances, 90% are covered by the top 623 chemicals, and 99% are covered by the top 2142 chemicals.

73 substances explain only 25% of all study-substance combinations recorded. They were mainly detected using targeted workflows (Figure S10). 137 substances cover 75%, 623 substances cover 90%, and 2142 cover 99% of all the study-sample combinations. The major substances include *ortho*-phthalates, lead, brominated and phosphor flame retardants, cadmium and other metal(loids) with 29–76 studies each (see Figure S10), mostly found in targeted analyses. The 73 most often detected substances in non-targeted workflows are displayed in Figure S11, with a median number of studies of about 10.

Table S6: Major substances according to the number of studies (n) by type of analysis (targeted vs non-targeted), as displayed in Figure 2 – A in the main manuscript. Note, that non-targeted approaches collected for this database were based on GC-HRMS and LC-HRMS and thus cover a limited chemical space (i.e. they do not cover all the potential chemical components, particularly excluding high-molecular-weight substances and metals).[S8] GC = Gas chromatography, LC = Liquid chromatography, HRMS = High-resolution mass spectrometry, CASRN = Chemical Abstract Service Number, BFRs = Brominated Flame retardants, PBDEs = Polybrominated Diphenyl Ethers.

Chem_id	Substance	CASRN	category	n
	**Targeted substances**			
435	Di(2-ethylhexyl) phthalate	117-81-7	Phthalates	76
3584	Lead	-	Metals	64
430	2,2',3,3',4,4',5,5',6,6'-Decabromodiphenyl ether	1163-19-5	BFRs - PBDEs	61
3570	Cadmium	-	Metals	55
3552	Bromine	-	BFRs	48
3236	Dibutyl 1,2-benzenedicarboxylate	84-74-2	Phthalates	47
3573	Chromium	-	Metals	44
3565	Antimony	-	Metals	43
3148	3,3',5,5'-Tetrabromobisphenol A	79-94-7	BFRs	34
3253	Benzyl butyl phthalate	85-68-7	Phthalates	34
3607	Zinc	-	Metals	34
3234	Diisobutyl phthalate	84-69-5	Phthalates	33
3233	Diethyl phthalate	84-66-2	Phthalates	32
3588	Mercury	-	Metals	32
3591	Nickel	-	Metals	31
3576	Copper	-	Metals	30
2866	2,2',4,4',5,5'-Hexabromodiphenyl ether	68631-49-2	BFRs - PBDEs	30
3344	Brominated diphenyl ethers	-	BFRs - PBDEs	30
3566	Arsenic	-	Metals	29
1207	2,2',3,4,4',5',6-Heptabromodiphenyl ether	207122-16-5	BFRs - PBDEs	29
	**Non-targeted substances**			
596	Butylated hydroxytoluene (BHT)	128-37-0	Antioxidants	30
3500	2,4-Di-tert-butylphenol	96-76-4	Antioxidants	26
435	Di(2-ethylhexyl) phthalate	117-81-7	Phthalates	24
3236	Dibutyl phthalate	84-74-2	Phthalates	22
2957	2,6-Di-tert-butylbenzoquinone	719-22-2	UV Stabilizers	22
3196	7,9-Di-tert-butyl-1-oxaspiro[4.5]deca-6,9-diene-2,8-dione	82304-66-3	UV Stabilizers	20
3234	Diisobutyl phthalate	84-69-5	Phthalates	19
7	Styrene	100-42-5	Monomers	19
3233	Diethyl phthalate	84-66-2	Phthalates	18
87	Bis(2-ethylhexyl) adipate	103-23-1	Plasticizers	17
2454	Hexadecanoic acid	57-10-3	Other organics	17
6	Ethylbenzene	100-41-4	Other aromatics	17
465	Benzophenone	119-61-9	UV Stabilizers	16
2762	Irganox 1300	6386-38-5	Antioxidants	16
564	Nonanal	124-19-6	Other organics	15
268	1-Methylbenzene	108-88-3	Other aromatics	15
3088	Acetyl tributyl citrate	77-90-7	Plasticizers	15
926	3,5-Di-tert-butyl-4-hydroxybenzaldehyde (BHT-CHO)	1620-98-0	Antioxidants	14
14	Benzaldehyde	100-52-7	Other organics	14
1660	(9Z)-Octadec-9-enamide	301-02-0	Other organics	14

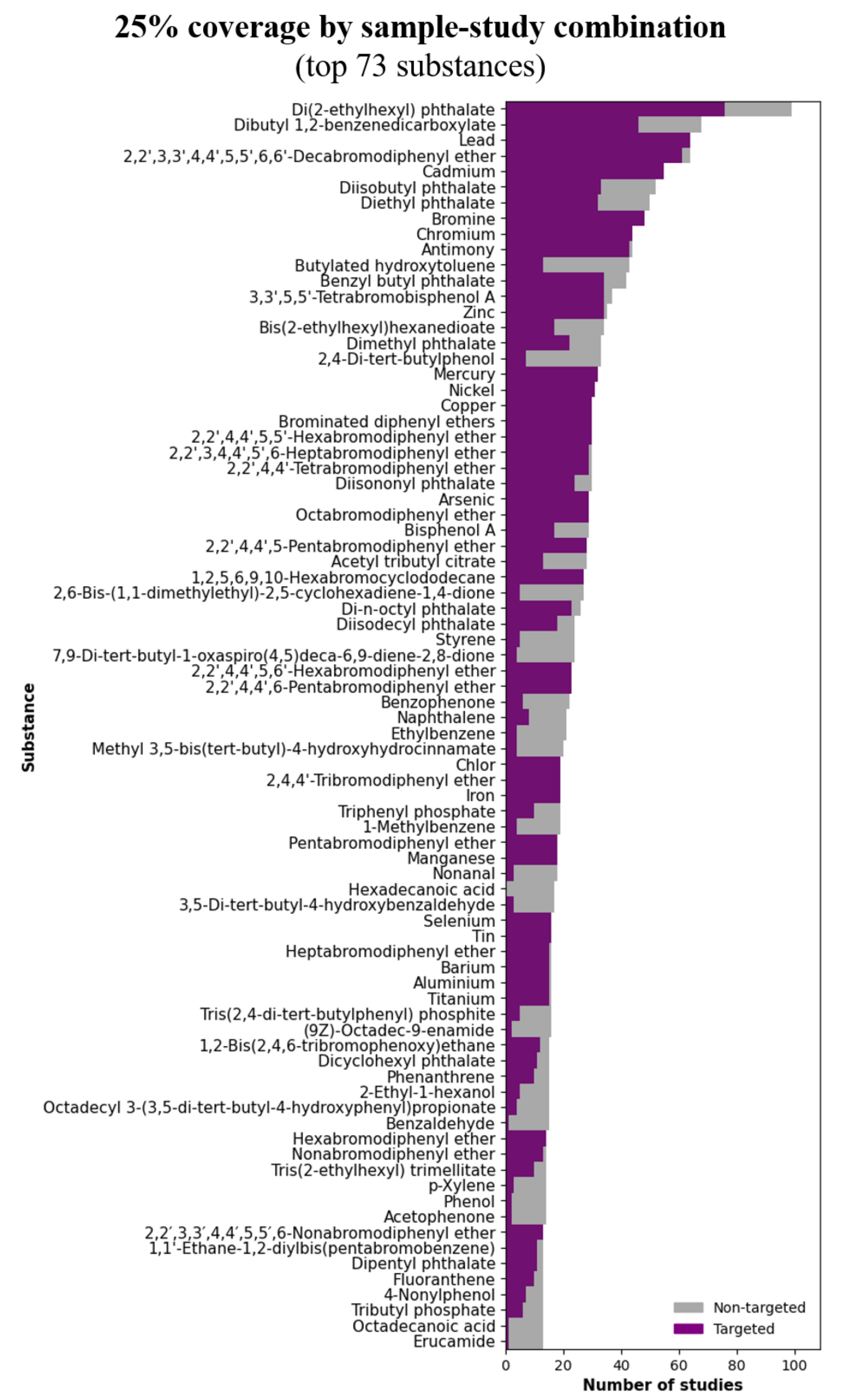

Figure S10: Top 73 substances covering 25% of all the sample-study combinations. Majority was part of targeted campaigns.

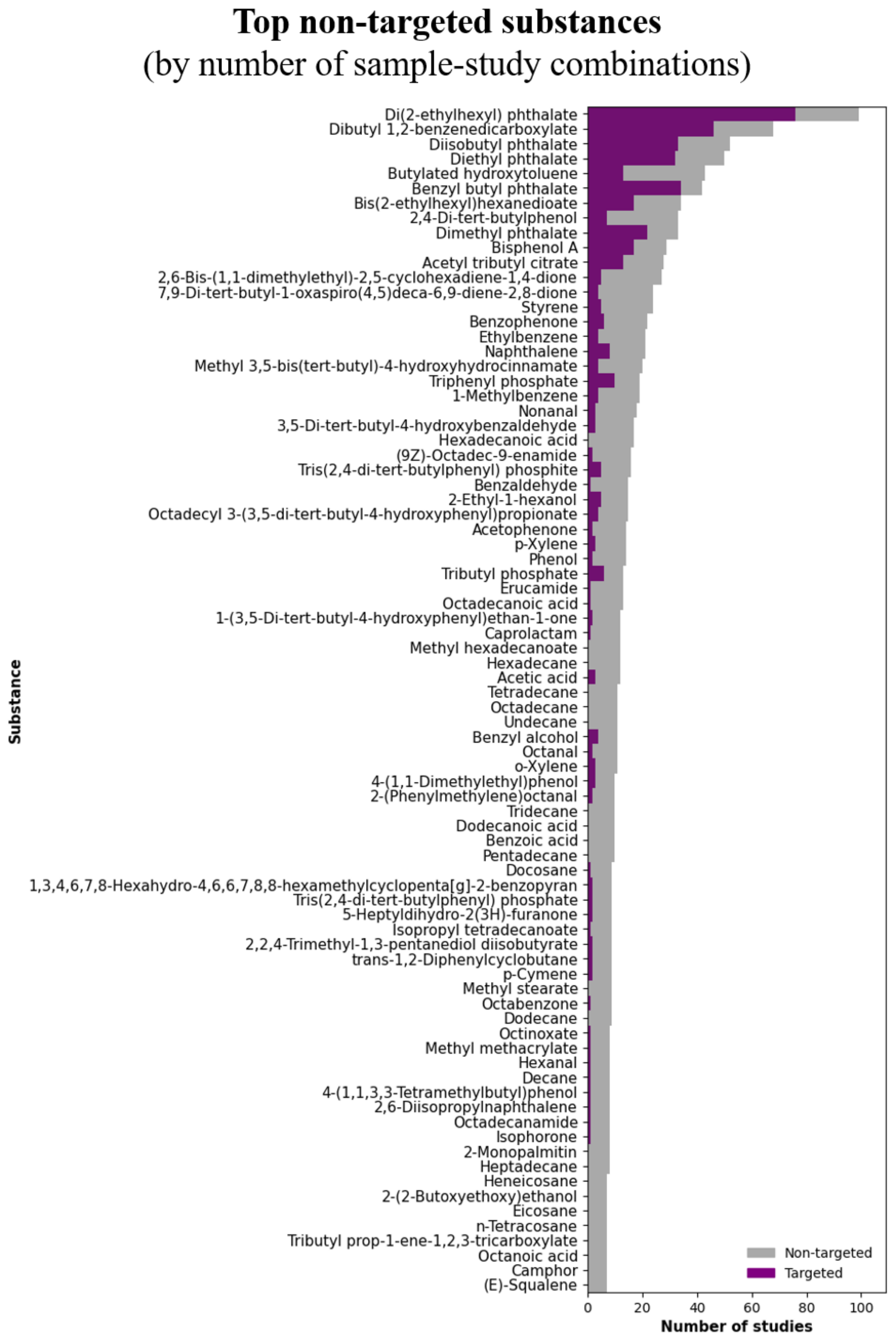

Figure S11: Top 73 non-targeted substances by the sample-study combinations.

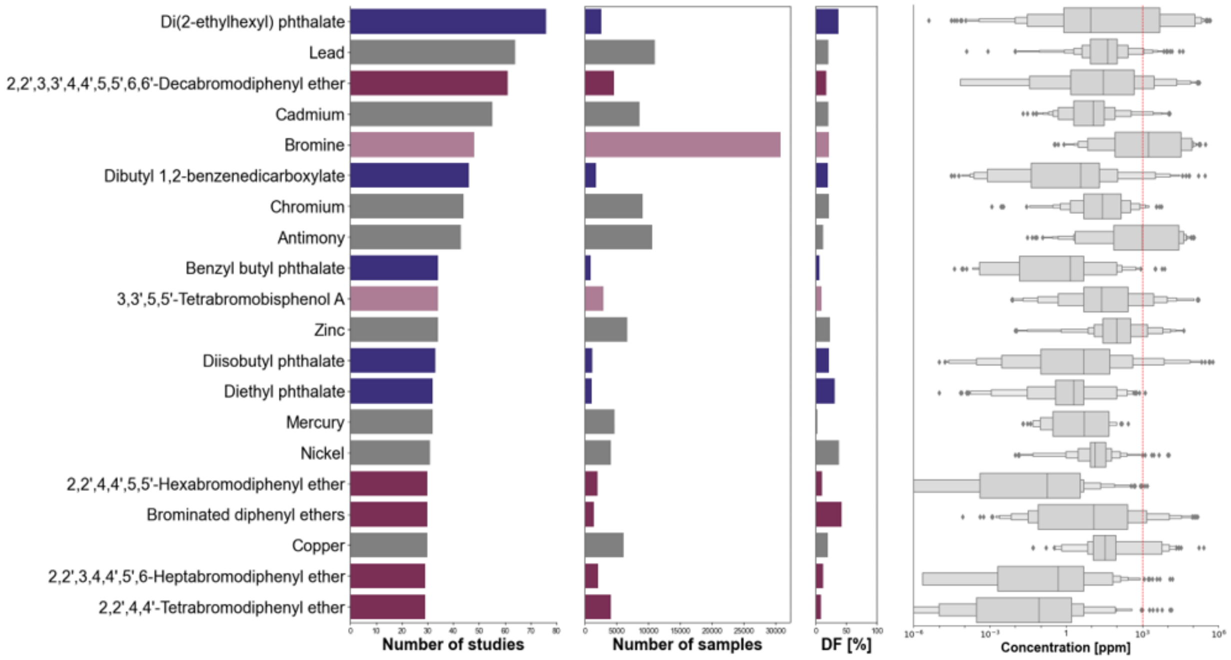

Figure S12: Top 20 targeted substances regarding the number of studies, the tested samples, their detection frequency (DF) and the measured concentrations.

Number of samples Few substances were detected in many samples (Figure S13). 45 substances explain 50% of all sample-substance combinations recorded in this study and were mainly detected using targeted workflows (Figure S14, 137 substances cover 75%, 623 substances cover 90%, and 2142 cover 99% of all the substance-sample combinations). The major substances include bromine, lead and antimony with 10 000–30 000 samples each (other major substances include other metal(loids), brominated and phosphor flame retardants, and ortho-phthalates, see Figure S14). Most of these substances were mainly found in targeted analyses. Furthermore, 739 substances (mainly from non-targeted analysis) were only detected in single samples and are thus need further confirmation regarding their relevance in plastics. The 45 most often detected substances in non-targeted workflows are displayed in Figure S15, and the median number of samples in which they were found is about 400.

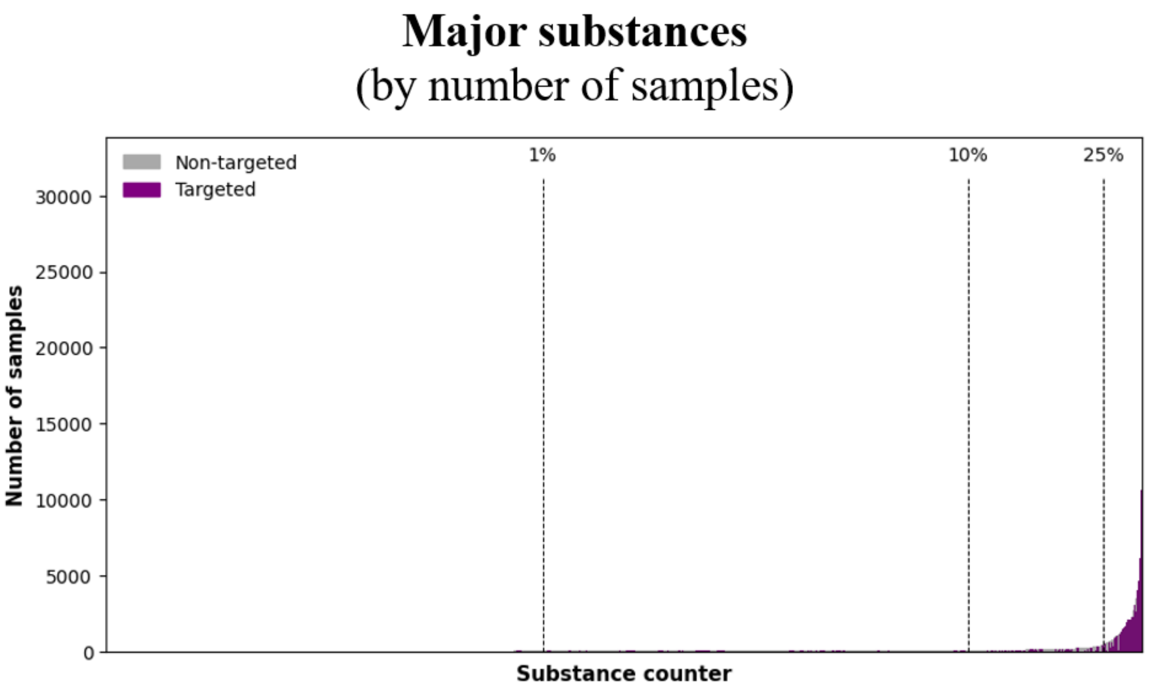

Figure S13: Major substances by the number of samples. 75% of the sample-substance combinations are covered by the top 137 substances, 90% are covered by the top 623 chemicals, and 99% are covered by the top 2142 chemicals.

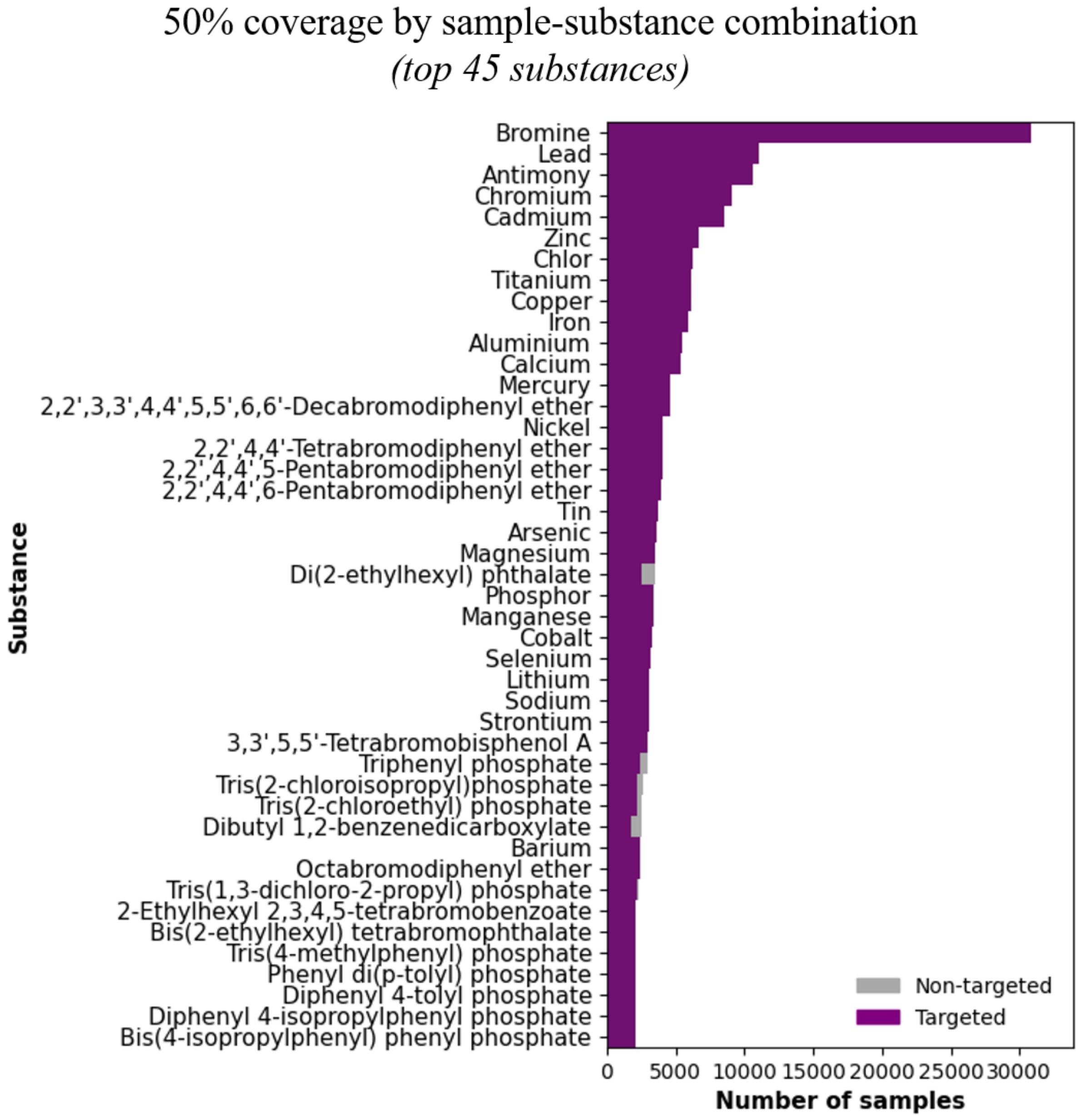

Figure S14: Top 45 substances covering 50% of all the sample-substance combinations. The majority was part of targeted campaigns.

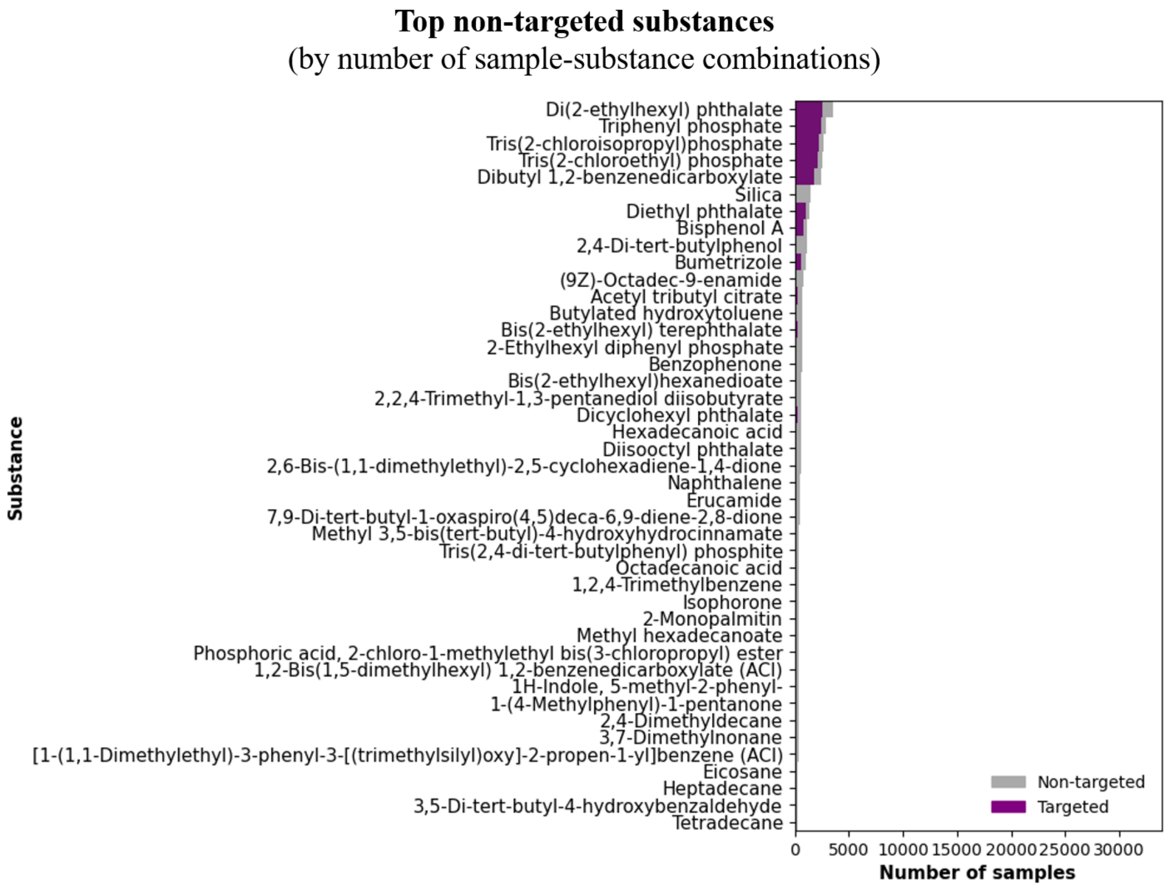

Figure S15: Top 45 non-targeted substances by the sample-substance combinations.

S2.2 Identification of gaps and research needs

Table S7: Overview of available data for different (sub-)product categories, as displayed in Figure 4 in the main manuscript.

**Category**	**Subcategory**	**Exposure potential**	**Global use [MT]**	**Number of** studies	samples	chemicals
**Packaging**	*Generic*	medium	142.60	108	6598	1615
	Food packaging	high	49.70	96	4434	1275
	Consumer packaging	medium	41.36	12	404	335
	Manufacturing packaging	low	23.25	1	4	5
	Hospitality packaging	high	12.97	0	0	0
	Retail packaging	low	9.01	0	0	0
	B&C packaging	low	3.87	0	0	0
	Agricultural packaging	low	2.45	0	0	0
**B&C**	*Generic*	low	76.89	34	664	541
	Thermal insulation	low	25.96	8	41	48
	Pipes	low	25.17	10	174	117
	Window Profiles	low	9.39	1	1	3
	Other	low	9.32	8	229	99
	Flooring	medium	5.97	15	158	404
	Roof lining	low	1.07	0	0	0
**Automotive**	*Generic*	medium	62.17	24	271	194
	Exterior	low	27.22	2	11	34
	Interior	high	27.22	8	69	69
	Tires	low	7.73	4	31	16
**EEE**	*Generic*	medium	17.31	82	29173	437
	ICT&CE	high	6.53	58	16700	265
	SHA	medium	4.99	19	1909	130
	CRAC	low	2.75	8	40	56
	LHA	low	2.70	13	1750	85
	Other	high	0.34	20	1387	202
**Agriculture**	*Generic*	low	3.78	3	56	121
	Silo Films	low	2.40	0	0	0
	Greenhouse Films	low	0.93	0	0	0
	Other	low	0.15	0	0	0
	Pipes	low	0.13	1	7	32
	Mulch Films	low	0.12	2	41	33
	Other Films	low	0.04	0	0	0
**Other**	*Generic*	medium	116.91	129	5633	1410
	Other	medium	59.78	9	30	172
	Medical	medium	17.65	47	971	432
	Household	high	12.54	47	1025	757
	Sports	medium	9.58	6	421	151
	Furniture	medium	9.04	8	143	173
	Toys	high	8.32	58	3043	840
**Textiles**	*Textiles*	medium	43.88	33	2788	886
	Apparel	high	12.33	12	170	231
	Other	low	10.64	1	1	11
	Household textiles	high	6.18	6	54	186
	Mobility textiles	medium	5.19	6	59	76
	Textile flooring	medium	4.81	7	45	449
	Furniture	medium	2.59	10	2296	404
	Agricultural nets	low	1.61	0	0	0

S2.2.1 Recyclability

Table S8: Factors for recycling-relevant case studies. MFA = Material flow analysis, OECD = Organisation for Economic Co-operation and Development.

	Property	exemplary data sources
**Product (sub)sectors**	high production, use, and waste amounts	use statistics,[S7] MFA studies[S6]
	high recycling potential (including material suitability and collectability)	MFA studies[S6,S9]
	common cross-sectoral recycling	MFA studies[S6,S9]
	long lifetimes	
	high exposure potential	
**Chemicals**	hazardous properties	OECD *eChemPortal*,[S10] PLASTICMAP database[S1]
	intentionally added in large amounts	plastic additive handbooks,[S11–S14] patents, production statistics[S15]
	commonly found in human or environmental samples	environmental or bio-monitoring studies
	available (quick) analytical techniques	SciFinder Analytical Methods[S16]

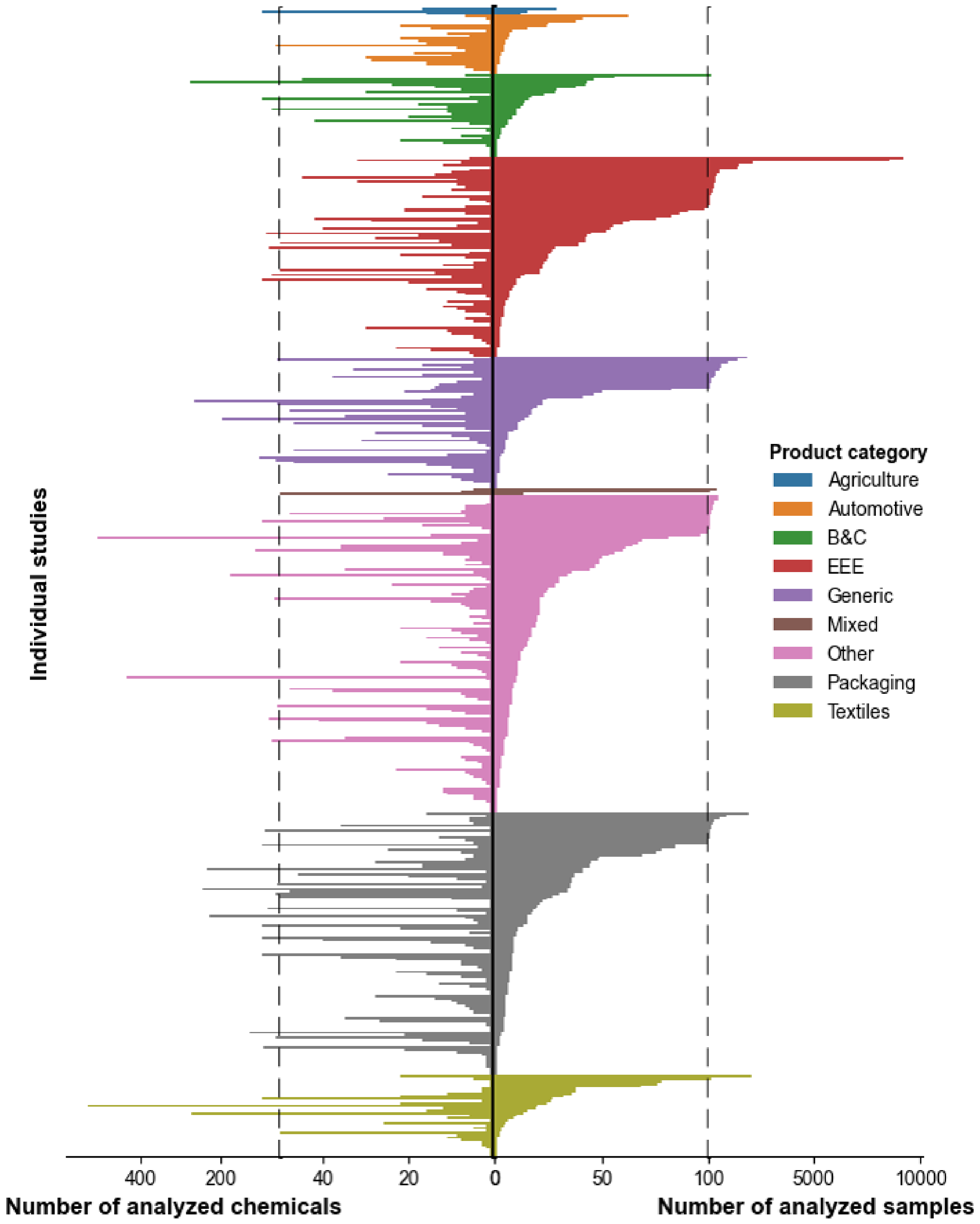

Figure S16: Number of chemicals and samples tested by individual studies, grouped by the product category (colors).

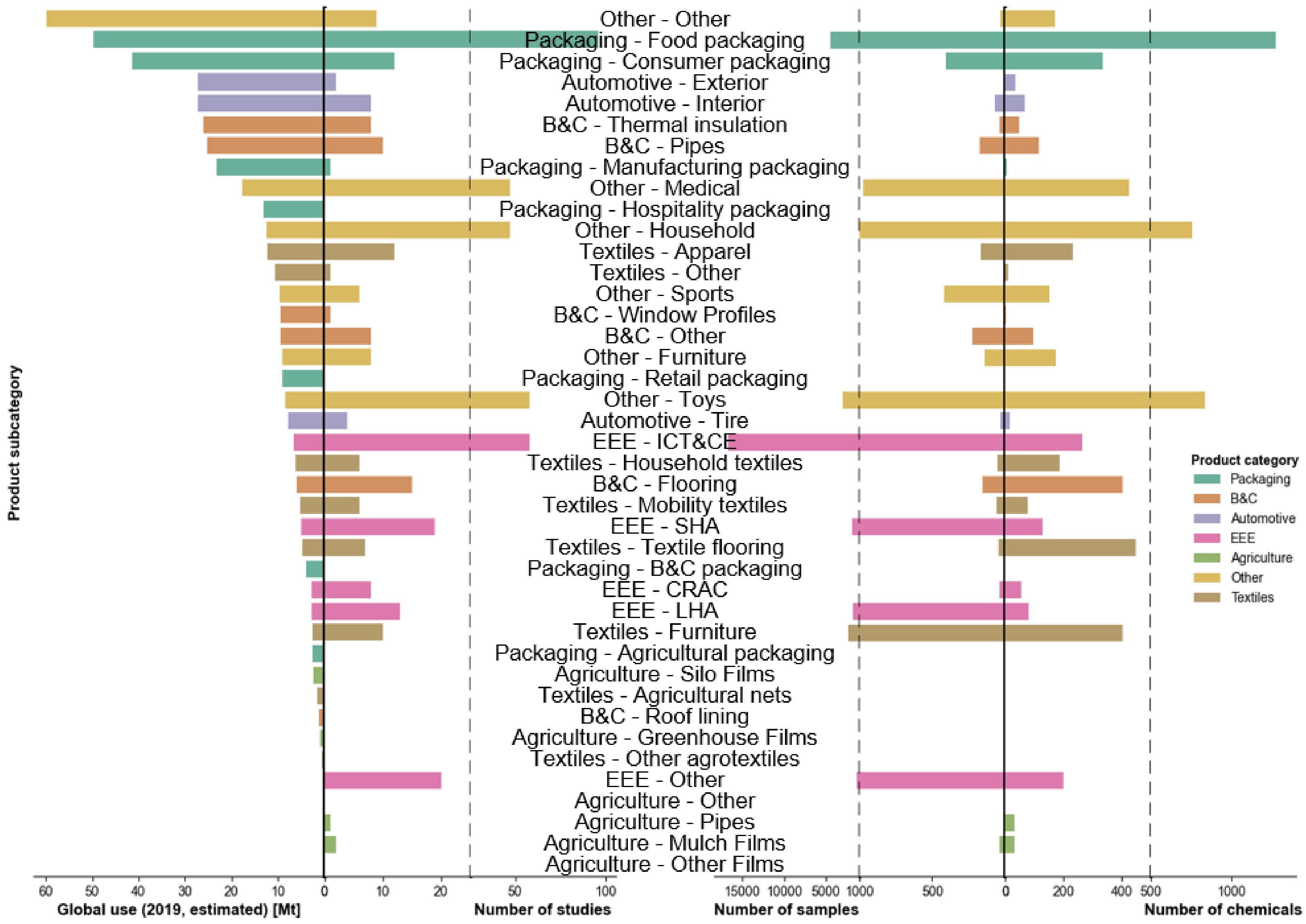

Figure S17: Global use and number of studies, samples, and chemicals by the product subcategory. Their global use per subcategory was extrapolated using the global use data by category and the Swiss subcategory use data, see Section S1.4.1.[S6,S7]

S2.2.2 Exposure potential

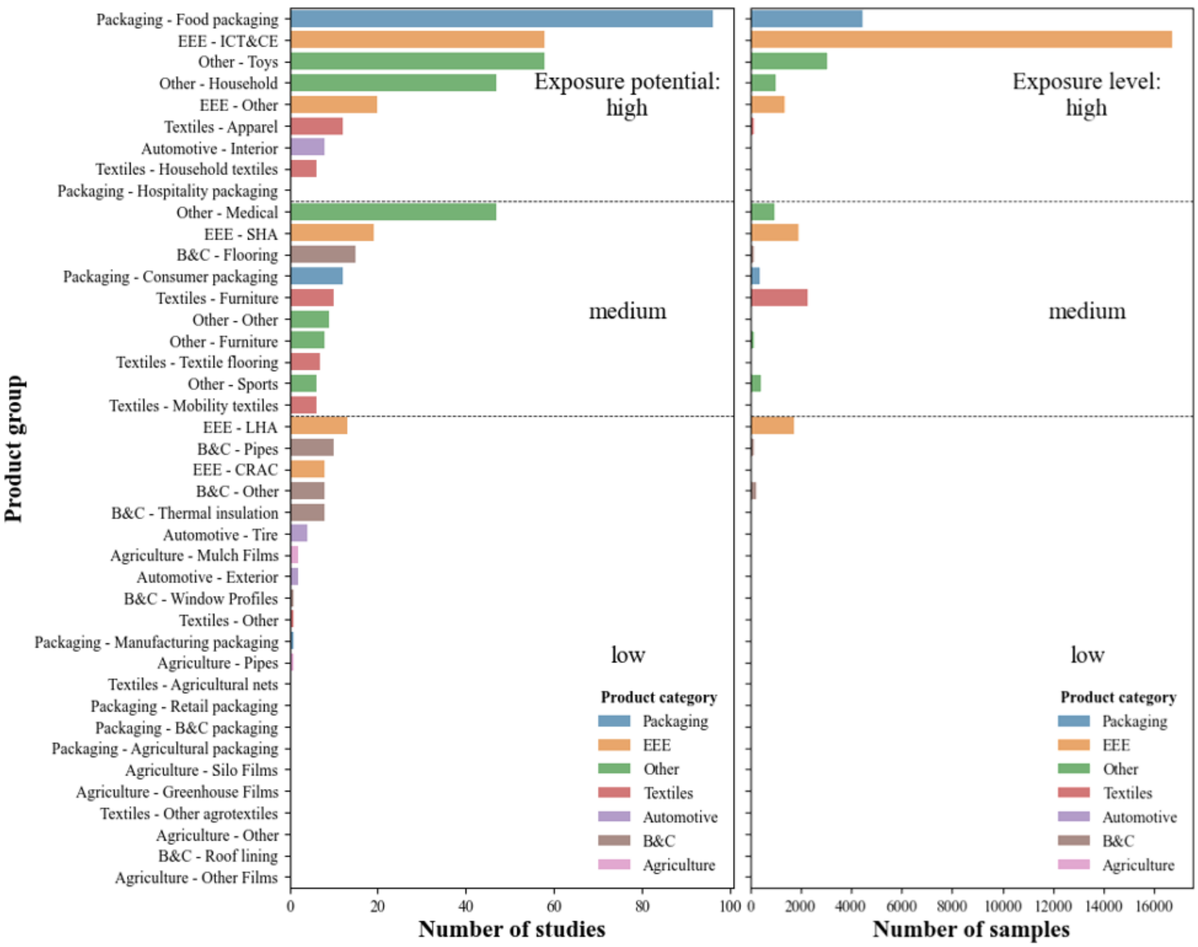

Figure S18: Number of studies (left) and samples (right) by the product subcategory, compared to their exposure potential.

S2.3 Potential effects of mechanical recycling

S2.3.1 Time-series of polybrominated diphenyl ether (PBDE) concentrations

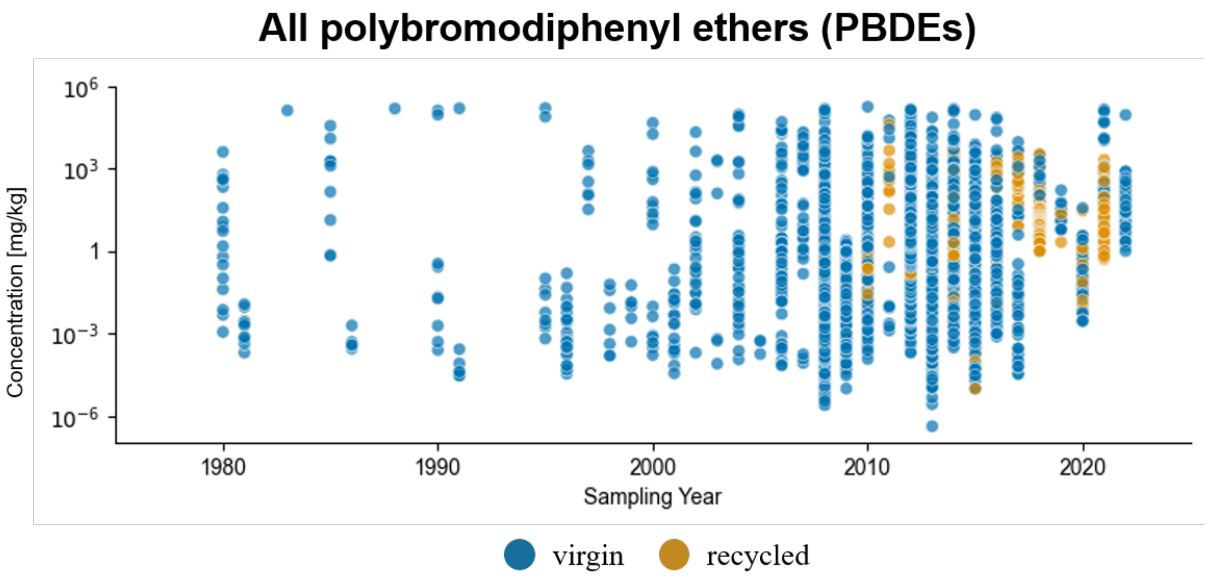

Figure S19: Concentrations of all measured PBDEs over time, distinguished by recycling status.

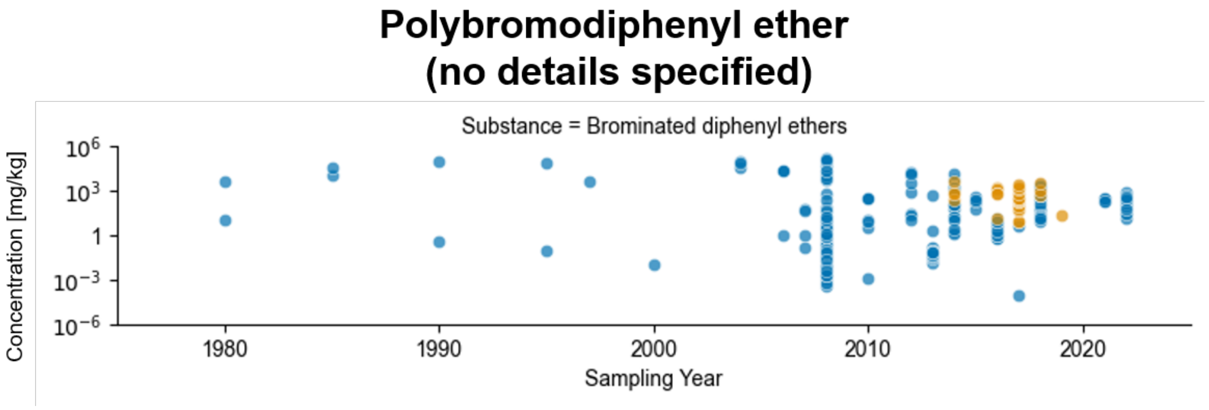

Figure S20: Concentrations of measured polybromodiphenyl ethers, that could not be assigned to any other subgroup, over time distinguished by recycling status.

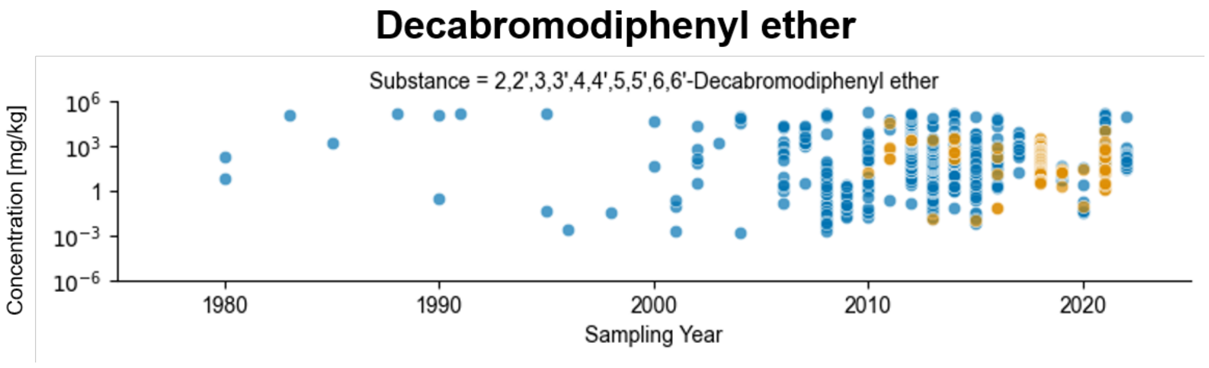

Figure S21: Concentrations of decabromodiphenyl ether (CASRN:1163-19-5) over time, distinguished by recycling status.

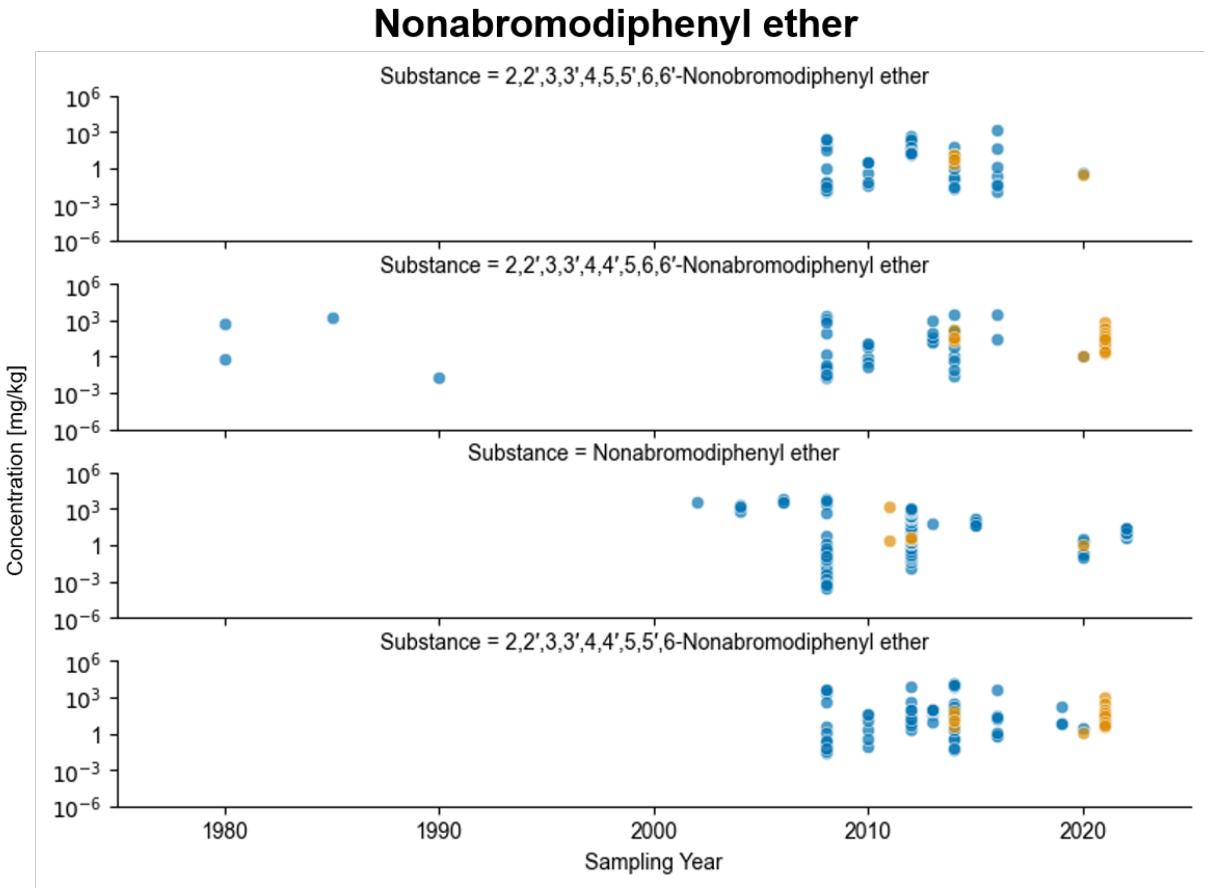

Figure S22: Concentrations of all measured nonabromodiphenyl ethers over time, distinguished by recycling status.

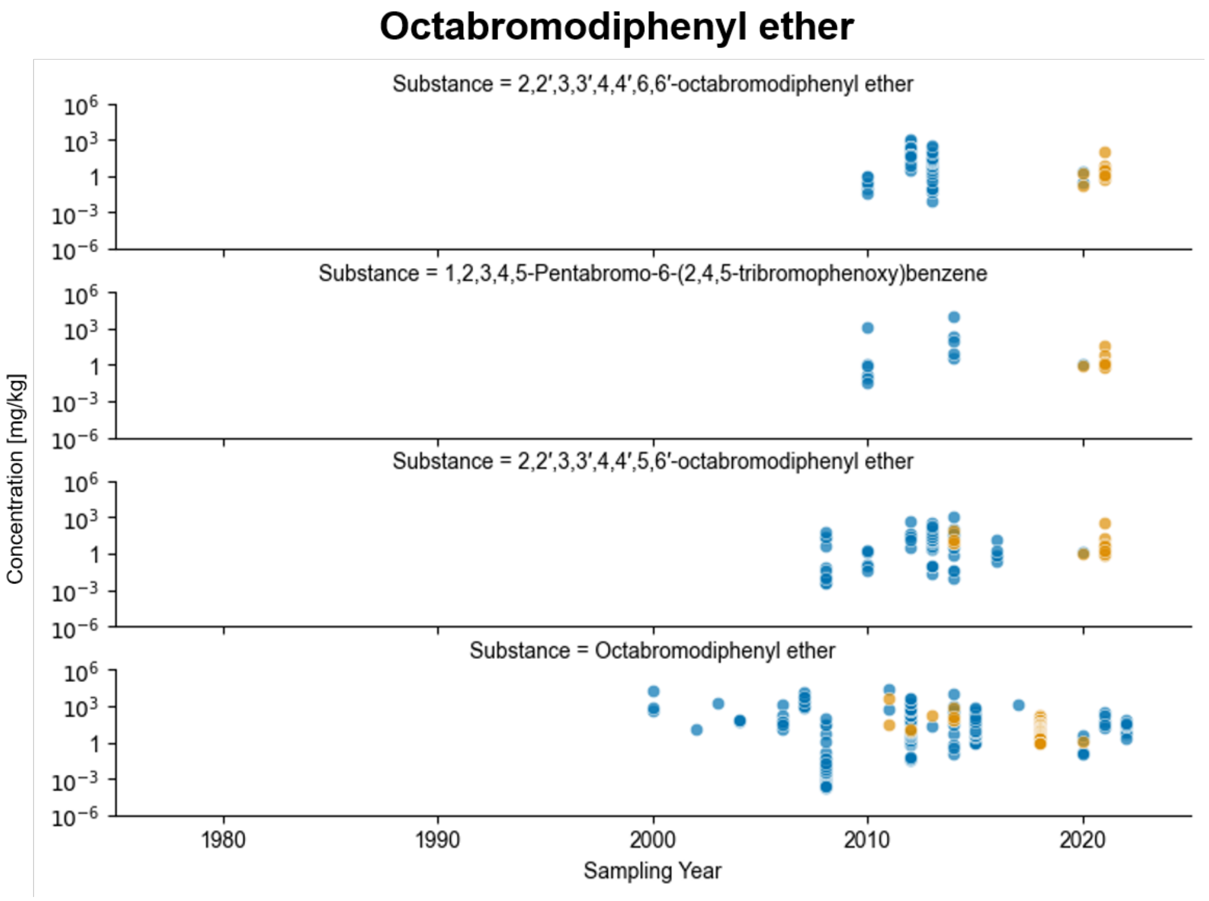

Figure S23: Concentrations of all measured octabromodiphenyl ethers over time, distinguished by recycling status.

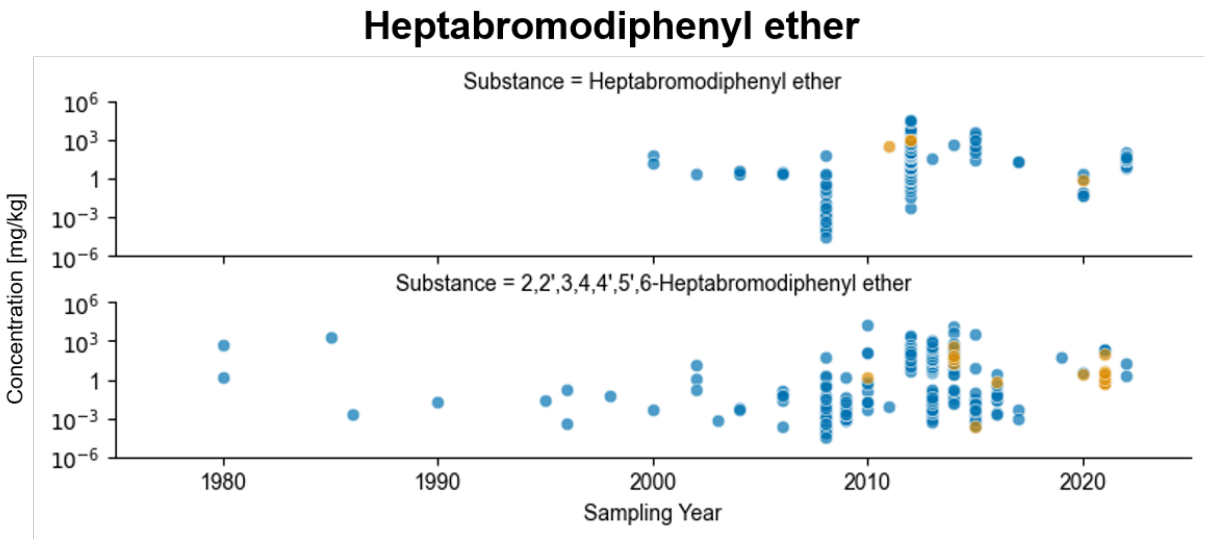

Figure S24: Concentrations of all measured heptabromodiphenyl ethers over time, distinguished by recycling status.

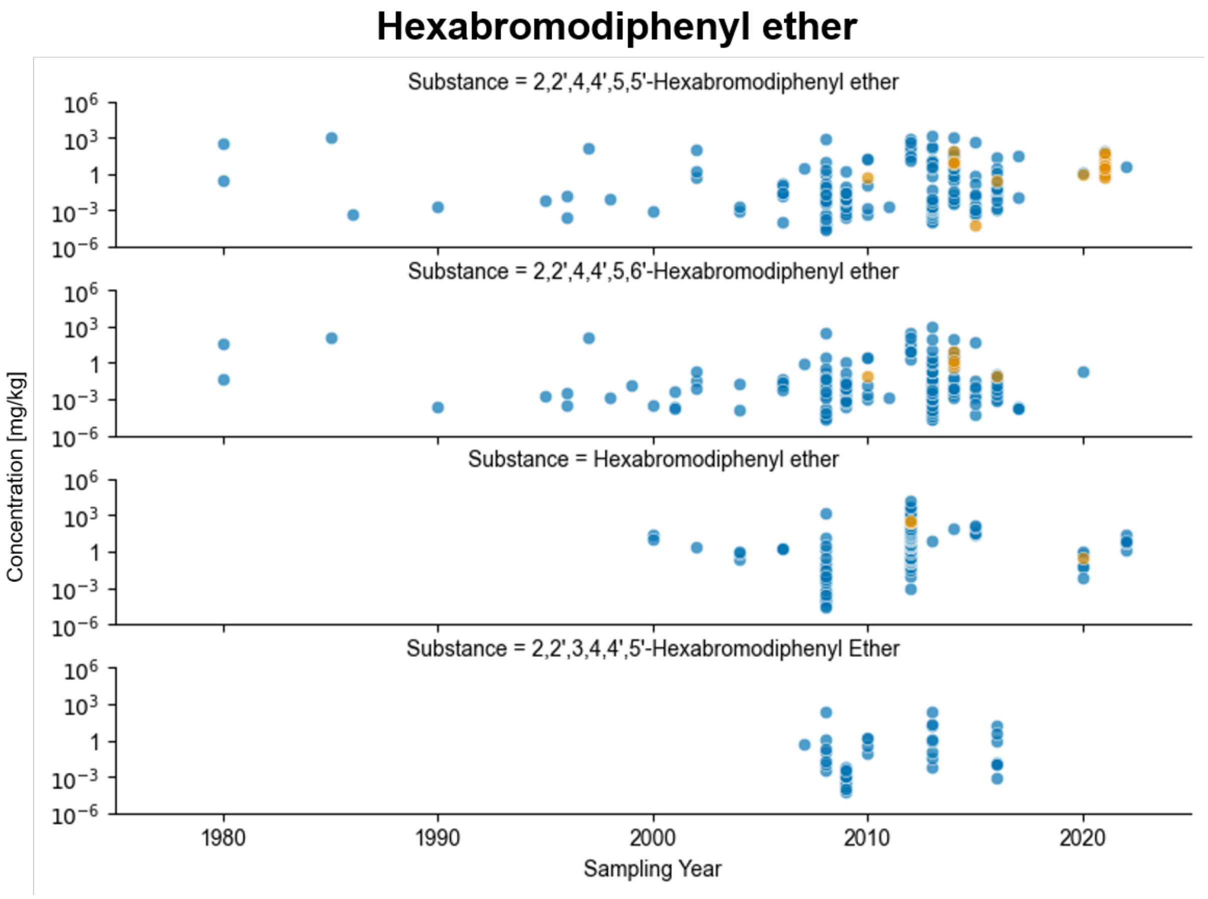

Figure S25: Concentrations of all measured hexabromodiphenyl ethers over time, distinguished by recycling status.

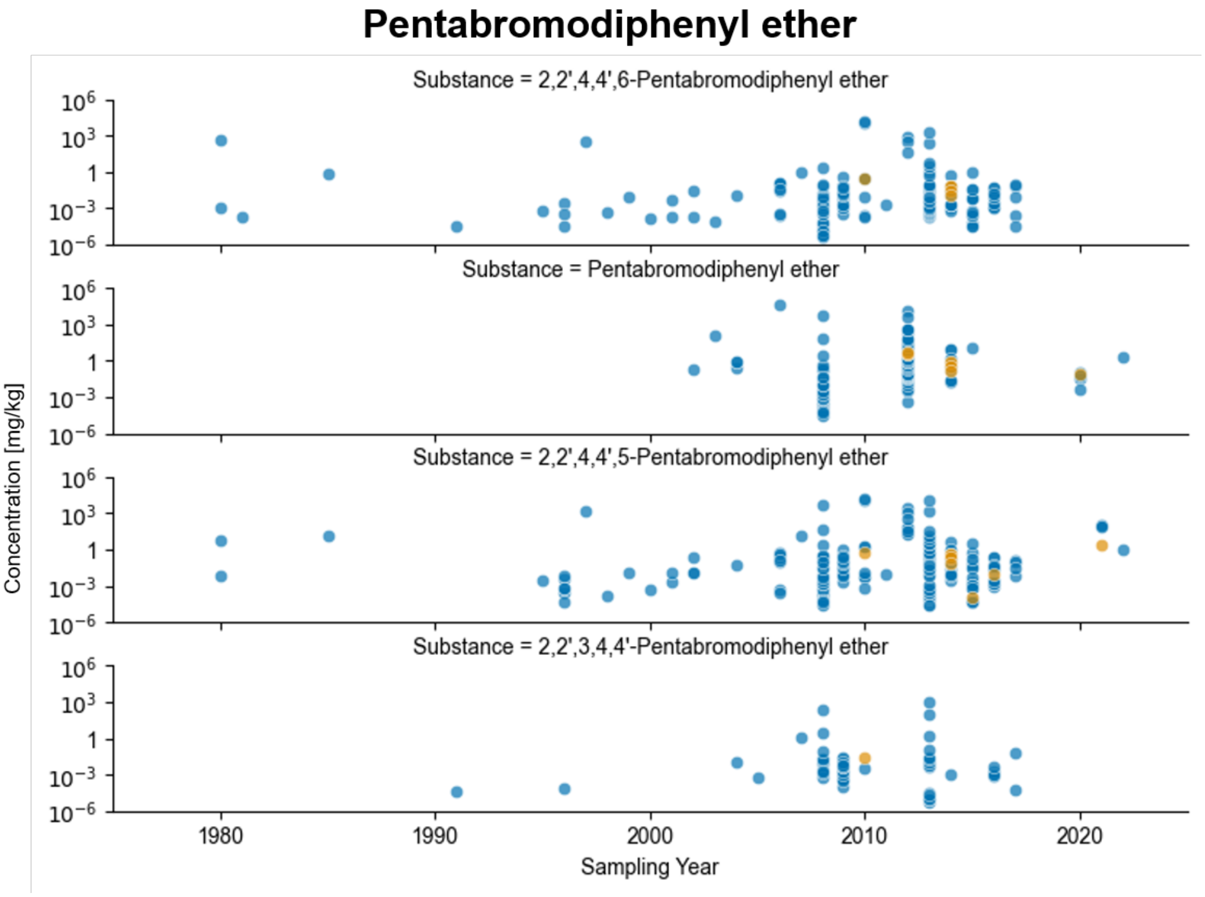

Figure S26: Concentrations of all measured pentabromodiphenyl ethers over time, distinguished by recycling status.

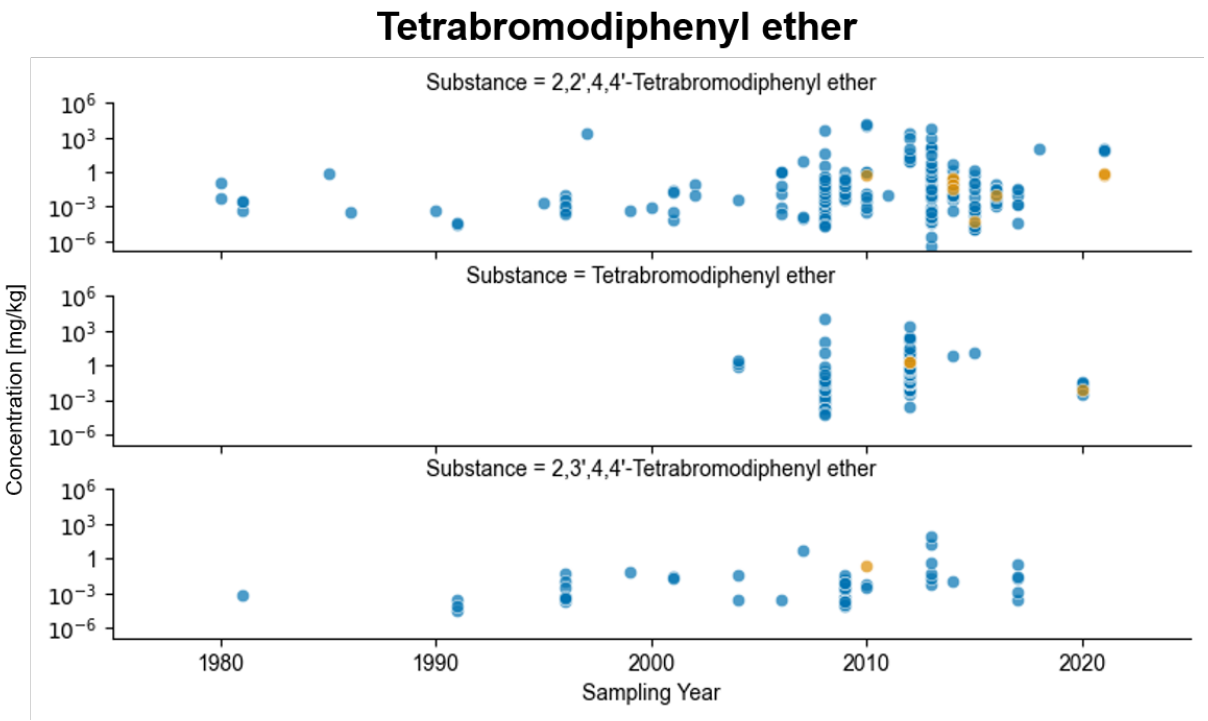

Figure S27: Concentrations of all measured tetrabromodiphenyl ethers over time, distinguished by recycling status.

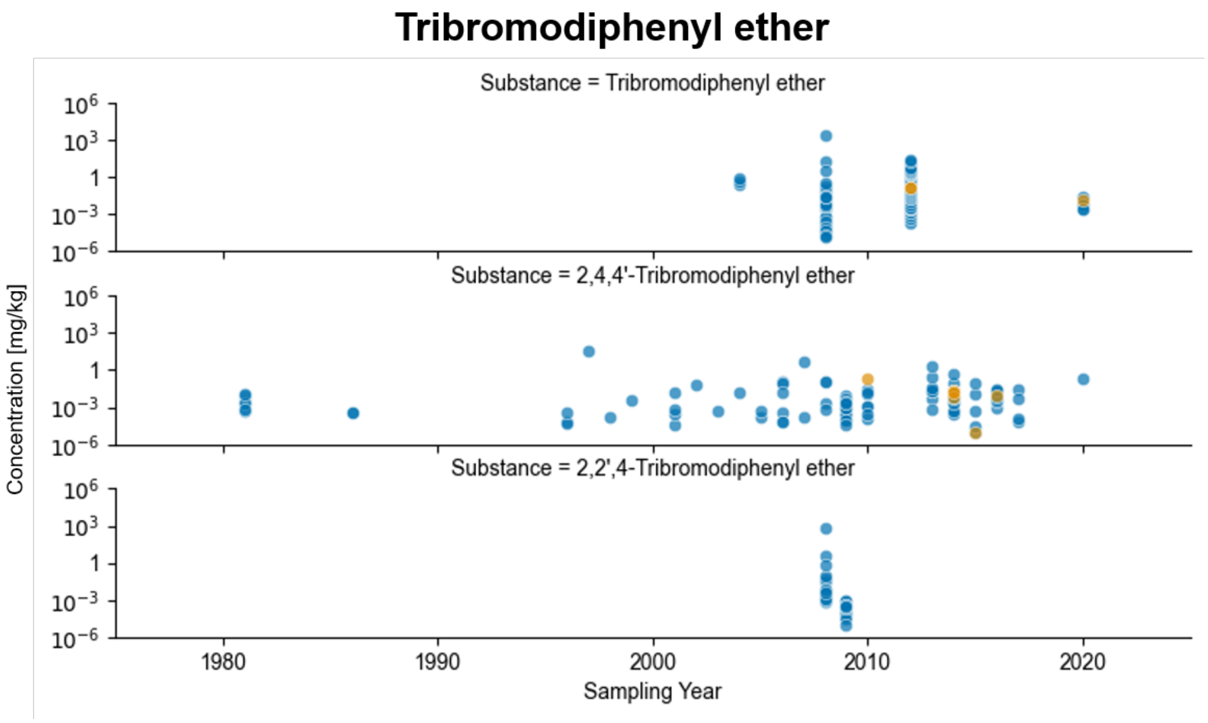

Figure S28: Concentrations of all measured tribromodiphenyl ethers over time, distinguished by recycling status.

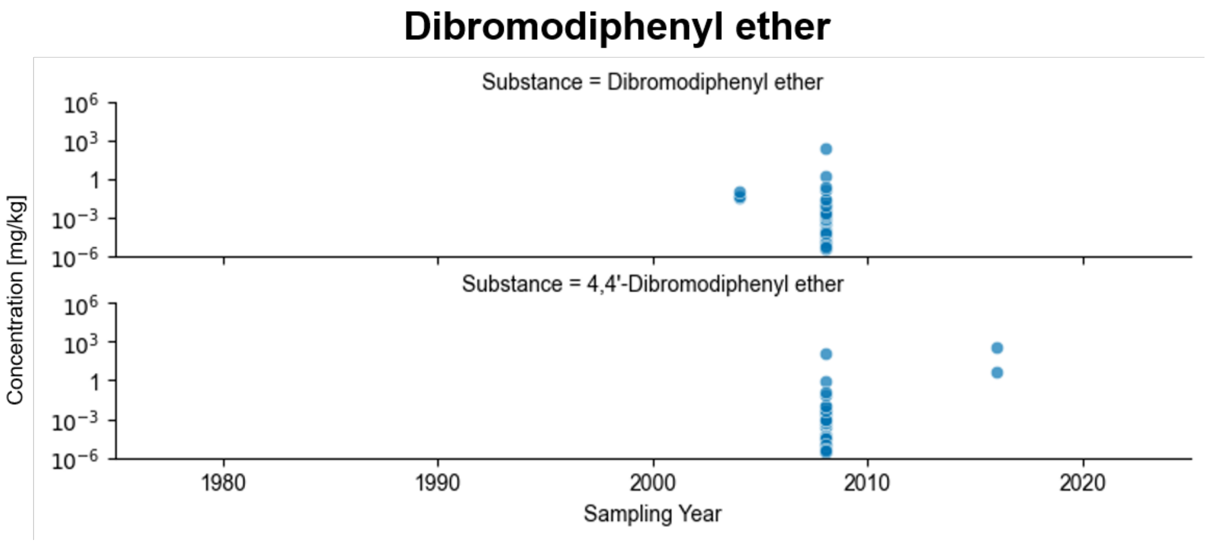

Figure S29: Concentrations of all measured dibromodiphenyl ethers over time, distinguished by recycling status.

References

(S1) Wiesinger, H.; Wang, Z.; Hellweg, S. Deep Dive into Plastic Monomers, Additives, and Processing Aids. *Environmental Science & Technology* **2021**, *55*, 9339–9351, DOI: 10.1021/acs.est.1c00976.

(S2) Web of Science; Clarivate Analytics Web of Science All Databases Help - Sort Options. 2024; https://images.webofknowledge.com/WOKRS521R5/help/WOK/hs_sort_options.html, accessed 2021-07-01.

(S3) Food packaging Forum (FPF) FCCmigex Database. 2023; https://www.foodpackagingforum.org/fccmigex, accessed 2024-04-30.

(S4) Jeon, S.; Kim, K. T.; Choi, K. Migration of DEHP and DINP into dust from PVC flooring products at different surface temperature. *Science of The Total Environment* **2016**, *547*, 441–446, DOI: 10.1016/J.SCITOTENV.2015.12.135.

(S5) Kawecki, D.; Scheeder, P. R. W.; Nowack, B. Probabilistic Material Flow Analysis of Seven Commodity Plastics in Europe. *Environmental Science & Technology* **2018**, *52*, 9874–9888, DOI: 10.1021/acs.est.8b01513.

(S6) Klotz, M.; Haupt, M.; Hellweg, S. Limited utilization options for secondary plastics may restrict their circularity. *Waste Management* **2022**, *141*, 251–270, DOI: 10.1016/j.wasman.2022.01.002.

(S7) Organisation for Economic Cooperation and Development (OECD) *Global Plastics Outlook*; OECD: Paris, France, 2022; p 201, DOI: 10.1787/de747aef-en.

(S8) Manz, K. E.; Feerick, A.; Braun, J. M.; Feng, Y.-l.; Hall, A.; Koelmel, J.; Manzano, C.; Newton, S. R.; Pennell, K. D.; Place, B. J.; Godri Pollitt, K. J.; Prasse, C.; Young, J. A. Non-targeted analysis (NTA) and suspect screening analysis (SSA): a

review of examining the chemical exposome. *Journal of Exposure Science & Environmental Epidemiology* **2023**, *33*, 524–536, DOI: 10.1038/s41370-023-00574-6.

(S9) Hsu, W.-T.; Domenech, T.; McDowall, W. How circular are plastics in the EU?: MFA of plastics in the EU and pathways to circularity. *Cleaner Environmental Systems* **2021**, *2*, 100004, DOI: 10.1016/j.cesys.2020.100004.

(S10) Organisation for Economic Cooperation and Development (OECD) Classification Search on eChemPortal. 2023; https://www.echemportal.org/echemportal/ghs-search, accessed 2023-10-05.

(S11) Bolgar, M.; Hubball, J.; Groeger, J.; Meronek, S. *Handbook for the Chemical Analysis of Plastic and Polymer Additives*, 2nd ed.; CRC Press: Boca Raton, 2015; DOI: 10.1201/b19124.

(S12) Gardner, W. In *Gardner's Commercially Important Chemicals*; Milne, G. W. A., Ed.; Wiley, 2005; DOI: 10.1002/0471736627.

(S13) Grossman, R. F. In *Handbook of Vinyl Formulating*; Grossman, R. F., Ed.; John Wiley & Sons, Inc.: Hoboken, USA, 2008; DOI: 10.1002/9780470253595.

(S14) Maier, R.-D.; Schiller, M. In *Handbuch Kunststoff Additive*, 4th ed.; Maier, R. D., Schiller, M., Eds.; Carl Hanser Verlag GmbH & Co. KG: München, 2016; pp I–XLVIII, DOI: 10.3139/9783446432918.fm.

(S15) Organisation for Economic Cooperation and Development (OECD) *Customisation Opportunities of IUCLID for the Management of Chemical Data – 3rd edition*; OECD Series on Testing and Assessment 297; OECD Publishing: Paris, France, 2023; DOI: 10.1787/4d380809-en.

(S16) Chemical Abstract Service (CAS) CAS Analytical Methods. 2024; https://methods.cas.org/, accessed 2024-01-08.
