# Supplementary figures and images for: LitChemPlast: An Open Database of Chemicals Measured in Plastics
Source: Environ Sci Technol Lett. 2024 Oct 29;11(11):1147–60. doi: 10.1021/acs.estlett.4c00355 (PMC11562724; doi:10.1021/acs.estlett.4c00355)

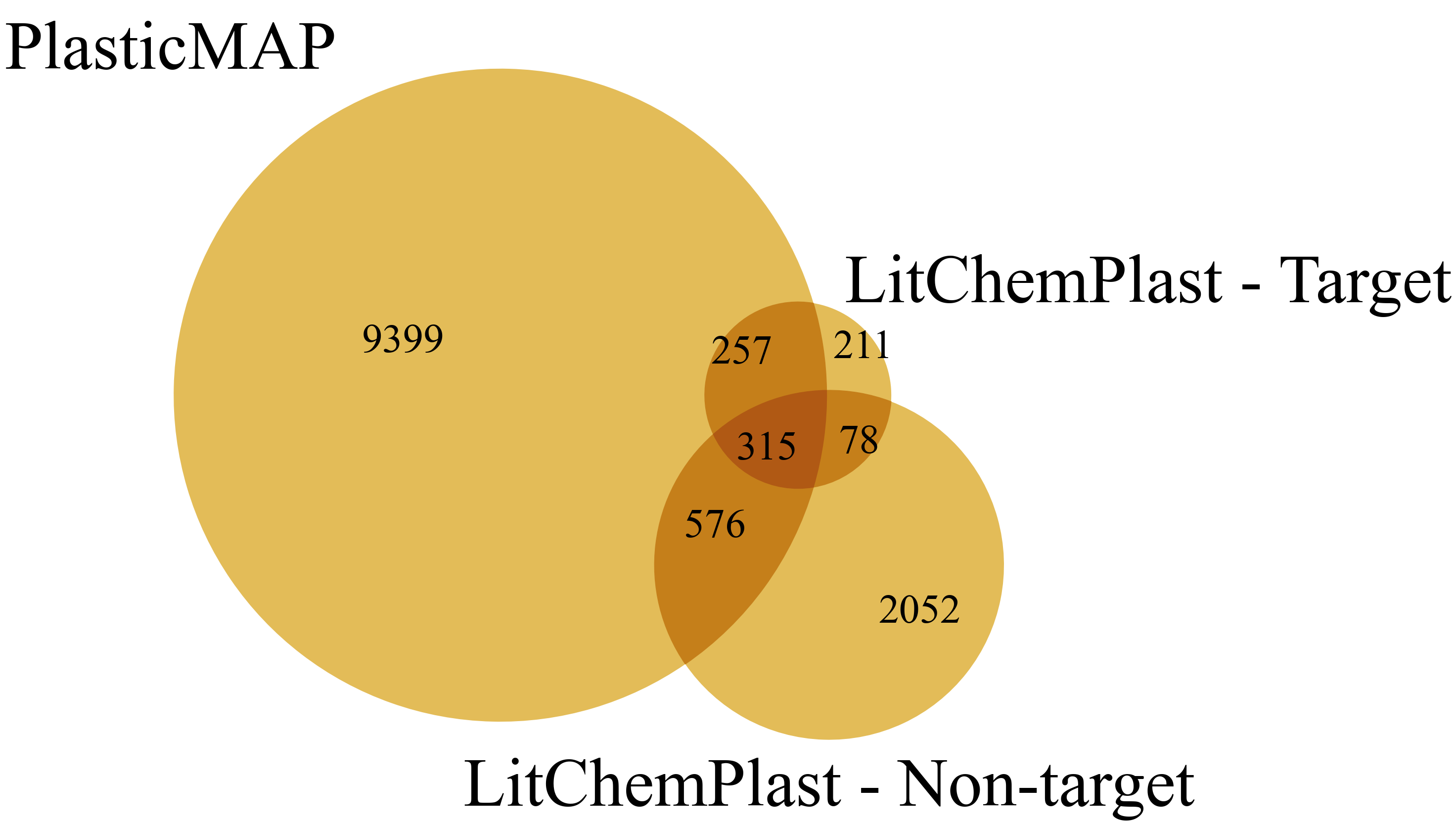

Supplement: Supplementary file 3 — ez4c00355_si_003.zip [file ez4c00355_si_003.zip › local_files_needed/Graphics/Venn_PlasticMAP-LitChemPlast - Target-LitChemPlast - Non-target.png]

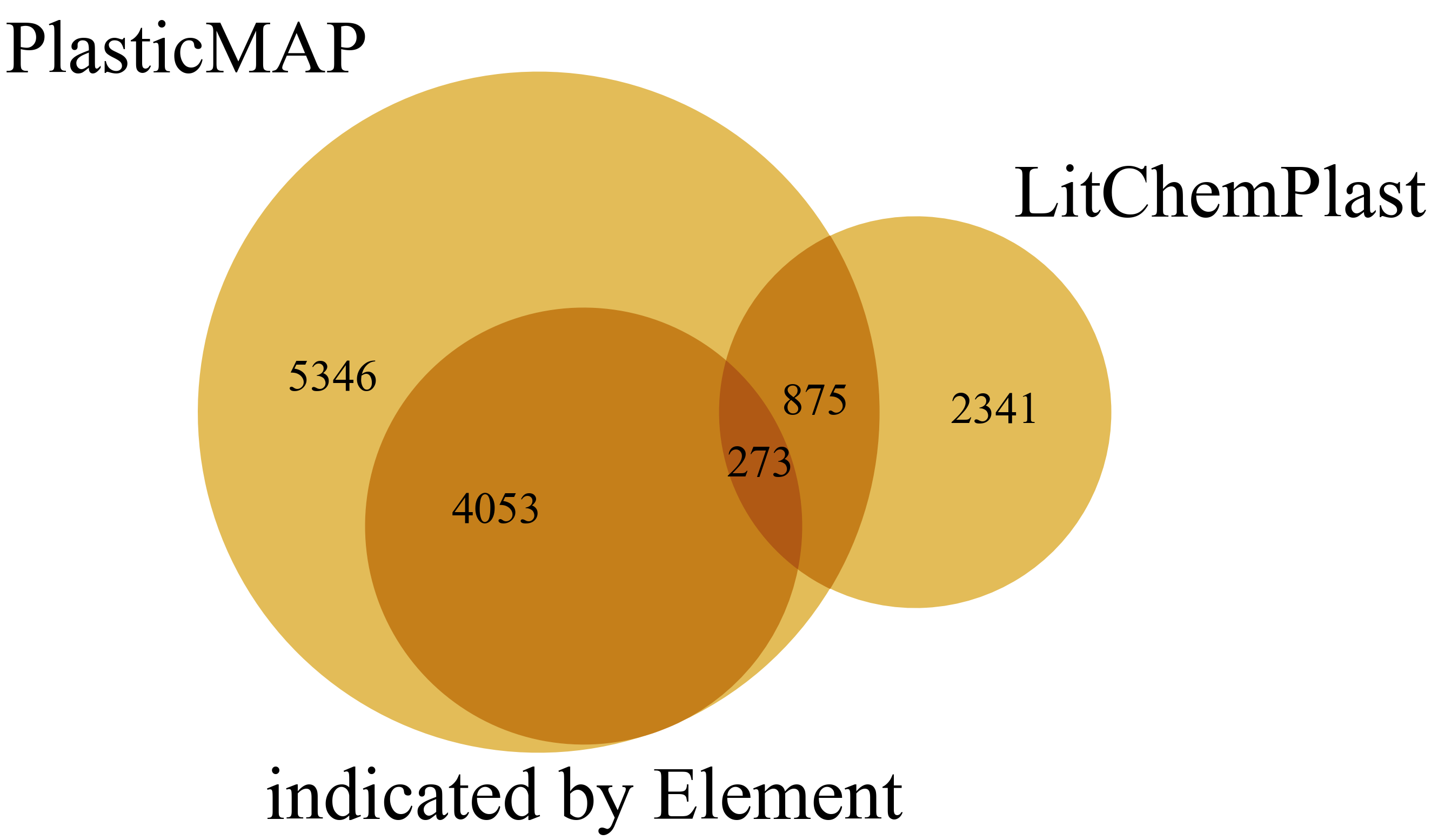

Supplement: Supplementary file 3 — ez4c00355_si_003.zip [file ez4c00355_si_003.zip › local_files_needed/Graphics/Venn_PlasticMAP-LitChemPlast-indicated by Element.png]
